# An annotated catalogue of salivary gland transcripts in the adult female mosquito, Ædes ægypti*

**DOI:** 10.1186/1471-2164-8-6

**Published:** 2007-01-04

**Authors:** José MC Ribeiro, Bruno Arcà, Fabrizio Lombardo, Eric Calvo, Van My Phan, Prafulla K Chandra, Stephen K Wikel

**Affiliations:** 1 Section of Vector Biology, Laboratory of Malaria and Vector Research, National Institute of Allergy and Infectious Diseases, National Institutes of Health, 12735 Twinbrook Parkway, Rockville, Maryland 20852, USA; 2 Department of Structural and Functional Biology, University 'FedericoII', Naples, Italy; 3 Parasitology Section, Department of Public Health, University 'LaSapienza', Rome, Italy; 4 Department of Immunology, School of Medicine, University of Connecticut Health Center, 263 Farmington Avenue, Farmington, Connecticut 06030, USA

## Abstract

**Background:**

Saliva of blood-sucking arthropods contains a cocktail of antihemostatic agents and immunomodulators that help blood feeding. Mosquitoes additionally feed on sugar meals and have specialized regions of their glands containing glycosidases and antimicrobials that might help control bacterial growth in the ingested meals. To expand our knowledge on the salivary cocktail of *Ædes ægypti*, a vector of dengue and yellow fevers, we analyzed a set of 4,232 expressed sequence tags from cDNA libraries of adult female mosquitoes.

**Results:**

A nonredundant catalogue of 614 transcripts (573 of which are novel) is described, including 136 coding for proteins of a putative secretory nature. Additionally, a two-dimensional gel electrophoresis of salivary gland (SG) homogenates followed by tryptic digestion of selected protein bands and MS/MS analysis revealed the expression of 24 proteins. Analysis of tissue-specific transcription of a subset of these genes revealed at least 31 genes whose expression is specific or enriched in female SG, whereas 24 additional genes were expressed in female SG and in males but not in other female tissues. Most of the 55 proteins coded by these SG transcripts have no known function and represent high-priority candidates for expression and functional analysis as antihemostatic or antimicrobial agents. An unexpected finding is the occurrence of four protein families specific to SG that were probably a product of horizontal transfer from prokaryotic organisms to mosquitoes.

**Conclusion:**

Overall, this paper contributes to the novel identification of 573 new transcripts, or near 3% of the *Æ. ægypti *proteome assuming a 20,000-protein set, and to the best-described sialome of any blood-feeding insect.

## Background

*Ædes ægypti *is a highly anthropophagic and cosmopolitan mosquito vector of epidemic dengue and yellow fever. To achieve fast blood feeding, adult female mosquitoes inject a complex salivary mixture into their hosts while probing for blood. Mosquito saliva, like that of other blood-feeding animals, has antihemostatic and antiinflammatory activities that counteract host responses that would otherwise restrict blood flow or call the attention of the host to the feeding site [1,2]. A preliminary transcriptome of adult female salivary glands (SG) has been previously reported [3] where 32 full-length transcripts have been described based on an analysis of 456 expressed sequence tags (EST). Of these putative proteins, ten have been verified by Edman degradation of Coomassie blue-stained bands from sodium dodecyl sulfate/polyacrylamide gel electrophoresis (SDS-PAGE) of SG homogenates. Most salivary proteins found have no known function.

The genome of *Æ. ægypti *has been recently made available, facilitating further gene discovery. In this paper, we present the analysis of an additional set of 3,776 SG cDNA sequences (total of 4,232 compared with previous set of 456 clones). We describe 573 new transcripts, 136 of which code for proteins of a putative secretory nature, most of which have no known function. We expect this work will contribute to the understanding of the evolution of blood feeding in arthropods and to the discovery of novel pharmacologic agents.

## Results and discussion

### General description of the salivary transcriptome database

A total of 4,232 clones was included in the EST salivary database, including 456 previously described [3]. The average length of the sequences was 752 bp, with 1419 sequences containing a polyA signature (20 contiguous A's). These clones assembled into 1,273 clusters (containing 2–261 sequences per cluster) and singletons (956 sequences). In this paper, we will use the word 'contig' to refer to clusters of one or more sequences. Mitochondrial sequences, identified by their match to sequenced *Aedes albopictus *and anopheline mitochondrial genomes, accounted for 73 EST from 13 clusters (thus far, the mitochondrial genome of *Æ. ægypti *is unknown). Of interest, several of these mitochondrial sequences mapped to scaffolds named supercont1.593 and also to supercont1.600, supercont1.929, and supercont1.363. Upon close inspections, these genomic scaffolds contain large segments of high similarity to *Ae. albopictus *mitochondrial genome. Accordingly, these contigs may be assigned to the mitochondrial genome in the final genome assembly, or may represent translocation of *Ae. aegypti *mitochondrial genes to the nuclear genome. To attempt a functional classification of these unique sequences, we compared them with proteome databases by blastx and with protein motifs by rpsblast (see Methods). Following manual annotation of these contigs, which included assignment of known or putative functions to the translation products, they were further divided into four categories: secreted (Sclass) with 352 contigs and 2,723 sequences; housekeeping (Hclass) with 739 contigs and 1,264 sequences; transposable element (Tclass) with 5 contigs and 9 sequences; and a last category composed of contigs coding for proteins of unknown function (Uclass) with 177 contigs and 234 sequences. The unknown class may contain truncated transcripts mainly mapping to 3' untranslated regions of genes. Although the Sclass corresponds to only 27% of the contigs, it consists of 64% of all EST, reflecting the relatively low complexity and abundance of the secretory material of the SG, as indicated before [3].

### Transcribed transposable elements

Nine transcripts in our database possibly derive from transposable elements. Their translation products are similar to those of the sea urchin *Strongylocentrotus purpuratus*[4] and to Tc1-like transposase[5]. These transcripts may indicate active ongoing transposition activity in *Æ. ægypti *or, more likely, they may represent regulatory elements suppressing transposition of relatively recent genome invasions.

### Housekeeping gene products

Putative Hclass genes were further classified according to their possible function (Table 1). Results are available online and can be searched on the columns labeled "Class" and "Comments" (Additional File 1) [6]. More than 50% of the sequences in this class derive from transcripts associated with protein synthesis, energy metabolism, protein modification, and protein export. Transporters and signal transduction gene products are also highly represented in the library. EST matching transporter proteins were found for several V-type ATPase subunits, Na^+^+K^+ ^ATPases, Ca^++ ^ATPases, aquaporin, and several families of solute carriers. V-type ATPases have been implicated in the secretion of saliva in Diptera [7].

**Table 1:** Functional classification of housekeeping transcripts.

**Putative function**	**Sequences**	**(%)**	**Contigs**	**(%)**
Protein synthesis	343	27.1	127	17.2
Energy metabolism	202	16.0	87	11.8
Protein modification	133	10.5	78	10.6
Protein export machinery	93	7.4	56	7.6
Signal transduction	102	8.1	69	9.3
Carriers, Transporters, Channels	64	5.1	49	6.6
Transcriptional machinery	40	3.2	31	4.2
Proteasome machinery	30	2.4	22	3.0
Nuclear regulation	26	2.1	23	3.1
Cytoskeletal	22	1.7	18	2.4
Amino acid metabolism	25	2.0	23	3.1
Intermediate metabolism	16	1.3	15	2.0
Oxidant metabolism	16	1.3	11	1.5
Transcription factors	16	1.3	14	1.9
Lipid metabolism	14	1.1	14	1.9
Carbohydrate metabolism	11	0.9	9	1.2
Nucleotide metabolism	6	0.5	5	0.7
Unspecified membrane proteins	4	0.3	3	0.4
Detoxication	3	0.2	3	0.4
Lysosomal enzymes	4	0.3	4	0.5
Unknown conserved	94	7.4	78	10.6
				
	1264		739	

### Updated catalogue of salivary proteins

We used the transcriptome set (Additional File 1) [8] and the Artemis tool to identify novel proteins coded in the *Æ. ægypti *genome. We have also assembled our transcripts with a set of ~220,000 EST of *Æ. ægypti *available in public databases to obtain eventual full-length information of translation products. This assembled EST dataset, similarly organized as that for AnoXcel [9], is available online [10]. Using these tools, we identified 614 protein sequences, mostly full length, deriving from the salivary transcriptome of adult female *Æ. ægypti *mosquitoes. Of these 614 protein sequences, 573 were identified and contributed to GenBank from this work, and the remaining were previously known. Of these 614 proteins, we identified a set of 136 putative secreted proteins expressed in the SG of *Æ. ægypti*, 97 of which are novel (Additional File 2) [11].

To obtain additional information potentially useful to address future functional analysis, we determined the tissue and sex specificity of a selected subset of 73 transcripts encoding secreted proteins and corresponding to a total of 71 genes. These 73 transcripts were selected based on their similarity to transcript families found in diverse mosquito species, and in the presumption that their translated products might play a role either in sugar or blood feeding. Oligonucleotide primers suitable for amplification of corresponding mRNA were employed for reverse transcriptase-polymerase chain reaction (RT-PCR) amplifications using as template total RNA extracted from female SG, female carcasses (i.e. adult females from which SG had been dissected), and whole adult males. Primers amplifying the ribosomal protein S5 were used for normalization and as control. The results of this analysis are summarized in Table 2. We previously used a similar assay for the analysis of the *Anopheles gambiæ *salivary transcriptome [12] and obtained results overlapping very well with the information independently obtained by Marinotti *et al*. [13] using the Affymetrix microarray chip. The results reported here also fit well with the data obtained comparing salivary versus nonsalivary libraries (Additional File 2) [6,11]. With our assay, it is possible to distinguish three broad classes of genes. First, genes that are female SG specific or whose expression is enriched in the female glands: they are indicated as SG or ENR, respectively, in Table 2. Products encoded by these genes are likely to play some role in blood feeding, for example as antihemostatics or immunomodulators. Among those genes analyzed, 31 belong to this class; more precisely, 23 were female gland specific, and 8 were enriched in female SG. They include both genes with unknown functions and genes known from previous studies on other mosquito species to be involved in the acquisition of blood meals (see below). A second group is represented by genes expressed in female glands and in adult males, without any expression in female carcasses: these are identified as SG,M in Table 2. It is very likely that most of these genes are gland specific and expressed both in male and female glands. The corresponding products may be involved in sugar feeding, antimicrobial activity, or other gland functions; 24 transcripts are members of this group. Finally, the last class includes genes with ubiquitous expression, i.e. expressed at approximately the same level in the three tissues examined and indicated as Ubiq in Table 2. These genes most likely encode polypeptides involved in housekeeping functions: 16 of the transcripts analyzed belong to this group. The following is a detailed description of the full-length transcripts found in the SG of adult female *Æ. ægypti *and of their profile of expression.

**Table 2:** Classification of 71 selected genes encoding putative secretory products as determined by RT-PCR expression analysis on female salivary glands, whole male mosquitoes, and carcass of female mosquitoes

Protein name or type	Comments	SG	ENR	SG, M	Ubiq	#
**Secreted carrier-like proteins including D7 family**						
gi|61742027	D7s1	X				
gi|94468624	D7s2 allele	X				
gi|18568330	D7s3	X				#
gi|16225992	D7l1			X		
gi|118216	D7l2		X			
gi|94468424	D7s4	X				
						
**Secreted protease inhibitors**						
Serpins						
gi|94468342	Similar to FXa-directed anticlotting	X				#
gi|94469320	Salivary serpin	X				
gi|18568304	Serpin			X		#
Other protease inhibitors						
gi|94468720	Kazal-containing peptide				X	
gi|94468612	Cystatin				X	
						
**Enzymes**						
Nucleotidases						
gi|94469274	Second apyrase or 5' nucleotidase			X		
gi|21654712	Salivary purine nucleosidase			X		
gi|1703351	Salivary apyrase			X		
gi|18568326	Putative adenosine deaminase		X			
Serine proteases						
gi|94468658	Serine protease			X		#
gi| 94468410	Salivary chymotrypsin-like enzyme			X		
gi|18568306	Putative serine protease			X		
gi|94468372	Trypsin like			X		
						
**Immunity-related proteins**						
Lectins						
gi|94468370	Putative salivary C-type lectin	X				
gi|18568318	Putative C-type lectin	X				#
gi|94468698	C type lectin				X	
gi|94468694	C-type lectin				X	
Angiopoietin						
gi|18568298	Angiopoietin-like protein	X				#
gi|94468352	Angiopoietin-like protein splice variant	X				
Antimicrobial polypeptides						
gi|48256697	Defensin A1				X	
gi|18568310	Gambicin				X	
gi|18568288	Putative lysozyme			X		
gi|94468690	Putative salivary peptide with HHH domain			X		
Other						
gi|94468654	i23M allele				X	
gi|18568294	Gram-negative binding protein			X		
gi|94468610	Peptidoglycan recognition protein				X	
						
**Unknown function, secreted, and ubiquitous**						
Antigen 5 protein family						
gi|18568284	Antigen 5 member	X				
gi|18568278	Putative secreted protein			X		
gi|18568308	Antigen 5 family member			X		
Other secreted proteins found in non-bloodsucking insects						
gi|94468620	PAN/APPLE-like domains				X	
gi|94468538	Cysteine-rich venom-like protein			X		#
						
**Unknown function, found in hematophagous Diptera salivary transcriptomes**						
56.5 kDa						
gi|18568292	Putative 56.5 kDa secreted protein			X		
41-kDa mosquito family						
gi|94468350	Putative 41-kDa salivary secreted protein				X	#
30-kDa/GE-rich family						
gi|94468546	30-kDa salivary gland allergen variant 2	X				#
gi|18568322	Putative 30-kDa allergen-like protein	X				#
29-kDa family						
gi|94468416	Putative salivary protein		X			
Other salivary proteins found in hematophagous Diptera						
gi|94468392	Putative secreted protein				X	
gi|94468374	Similar to *An. gambiæ *gSG8 protein			X		
gi|61742023	Putative 8.9-kDa secreted protein			X		
gi|94468386	Putative 23.4-kDa salivary protein			X		
gi|94468986	Putative secreted peptide				X	
gi|94468460	Putative secreted peptide				X	
gi|94468614	Unknown secreted	X				
gi|94468634	Possible secreted protein	X				#
gi|94468408	Putative secreted salivary peptide				X	
						
**Aedes genus specific**						
62kDa family – Single exon						
gi|18568300	Putative secreted protein	X				#
gi|18568302	Putative secreted protein	X				#
34-kDa gene family						
gi|94468412	Salivary peptide similar to 34-kDa protein		X			
gi|94468650	Putative salivary peptide	X				
gi| 94468336	Putative 34-kDa secreted protein family	X				
gi|18568296	Putative 34-kDa secreted protein		X			
30.5-kDa						
gi|94468432	Putative 30.5-kDa secreted protein		X			
gi|61742033	Putative 30-kDa secreted protein	X				#
9-kDa family						
gi|94468426	Salivary peptide with WWW domain			X		
gi|94468396	Putative secreted peptide			X		
Other Aedes-specific polypeptides						
gi|94468530	Putative salivary secreted peptide				X	
gi|94468356	Putative salivary peptide with basic tail		X			
gi|18568314	Putative 18.6-kDa secreted protein			X		
gi|94468380	Putative 18.6-kDa secreted protein – variant			X		
gi|18568282	Putative 7.8-kDa secreted protein		X			#
gi|94468390	Putative basic peptide 4.2K-1	X				
gi|94468394	Proline-rich peptide	X				
gi|94468568	Putative salivary secreted peptide			X		
gi|42632615	Putative 8.7-kDa secreted protein	X				#
gi|94468646	Peptide similar to insulin-like growth factor				X	

The four classes are SG (signal found exclusively in adult female salivary glands); ENR (enriched in adult female salivary glands); SG,M (found in adult female salivary glands and in males, but not in carcasses of adult females lacking salivary glands); and Ubiq (genes ubiquitously found in all tissues tested). Clones marked with # were analyzed three times, all others twice. Clones 94468370, 48256697, and 94468634 may undergo alternative splicing. Although clones 94468546 and 18568322 (30 kDa), 94468614, 18568300, and 18568302 (62 kDa) were assigned to SG class, some faint bands were seen in some samples from males. Clones 94468620, 94468350, 94468634, and 42632615 (primer pairs 23, 3, 29, and 60) underwent 35 cycles due to low amplification levels.

### Secreted salivary proteins

#### Proteins with some function confirmed or presumed from structure

##### Secreted ligand carrier-like proteins including D7 family

###### D7 salivary proteins

The first D7-coding gene was reported 15 years ago, for the mosquito *Æ. ægypti *[14]. It was later found in virtually all mosquito sialotranscriptomes where short (~15 kDa) and long (~30 kDa) forms were recognized [15]. In *An. gambiæ*, this gene family, except for one poorly transcribed gene, appears to be selectively expressed in the female SG [17,18], indicating a role in blood feeding. This protein family is distantly related to the odorant-binding protein (OBP) family, which specializes in binding small ligands [17]. Recently, some mosquito D7 proteins were shown to bind and inhibit the action of biogenic amines such as serotonin, histamine, and norepinephrine, a function that might help blood feeding [18]. Additionally, one short D7 protein from *Anopheles stephensi*, named hamadarin, was shown to prevent kallikrein activation by FactorXIIa [19]. Long D7 forms also exist in sand flies [16] and Culicoides [20], indicating that this gene family was recruited very early in the evolution of hematophagous Nematocera.

**Figure 1:**
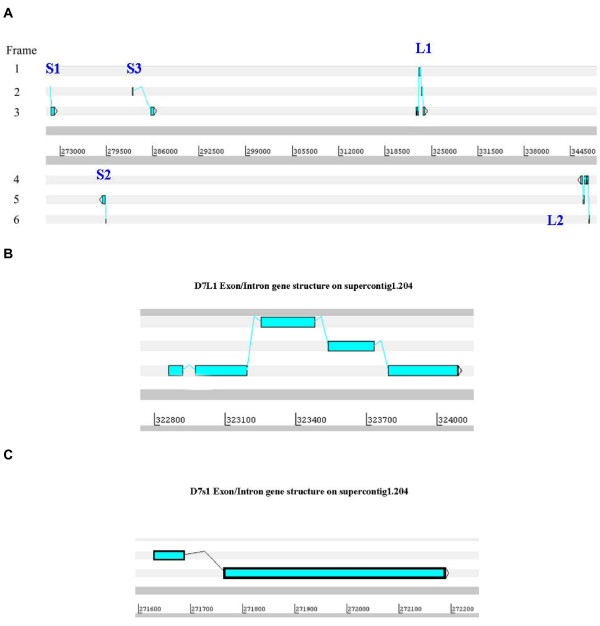
**The D7 gene cassette in supercontig1.204 of *Ædes ægypti***. **A**, Overview of genomic region containing three short and two long D7 genes. **B**, Exon-intron structure for gene *D7L1*. **C**, Exon-intron structure for gene *D7s1*.

In the mosquito *An. gambiæ*, five short and three long D7 proteins are known. Their genes are organized in a inverted tandem repeat[21] where the coding region for the three long proteins is followed by the five genes coding for the short protein in the reverse orientation [12]. In *Æ. ægypti*, three short and two long D7 proteins map to the assembled genome supercontig1.204 (Figure 1), and one short D7 protein maps to supercontig1.253 (not shown). The genomic region coding for the D7 proteins in supercontig1.204 shows three short *D7 *genes followed by two genes coding for long forms; however, while in Anopheles the frame orientation of the short and long forms is the same (but the short and long forms are in reverse orientation to each other), in Ædes there is no consistent orientation (Figure 1A). Similarly to *An. gambiæ*, all large Ædes *D7 *genes contain five exons (Figure 1B); however, all short Ædes D7 genes have two exons (Figure 1C), including that in supercontig1.253 (not shown), while in Anopheles, four of the five genes coding for short D7 proteins have three exons. The anopheline gene coding for the two-exon gene is poorly expressed, leading to the suggestion that this two-exon gene may be turning into a pseudogene [18]. The differences between Anopheles and Ædes in the number of D7 coding genes, their exon number, their orientation, and their chromosome location (in Anopheles, all eight genes are clustered, while one Ædes gene is far apart from the main cluster of five genes) are consistent with the ~150 million years of separation between culicines and anophelines [22].

**Figure 2:**
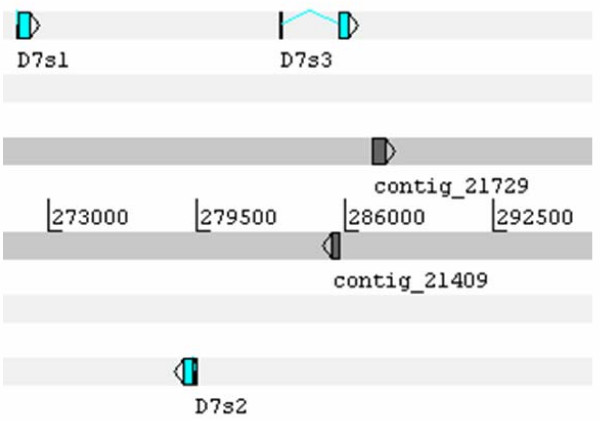
Additional transcripts observed mapping to the D7 gene region of *Ædes ægypti *on supercontig1,204.

In agreement with the larger genome size of Æde*s *[23], the five-gene D7 cassette of Ædes spans nearly 80 kb, four times more than the eight-gene cassette of Anopheles. To investigate whether additional genes were expressed within the *D7 *gene cassette in *Æ. ægypti*, we mapped ~220,000 EST to the *Æ. ægypti *genome. No new identifiable expressed genes were revealed in the D7 region; however, in the vicinity of the short D7 cassette, we found two transcripts, both deriving from SG libraries, which map to the intron of D7s3 and to the 3' region of the same gene (Figure 2). Translations of these two EST do not reveal extended open reading frames. This finding is reminiscent to the D7 short gene region of Anopheles, which also has an apparently noncoding EST mapping to the end of the short cassette, but at its 5' end. We have hypothesized previously that these noncoding transcripts could be associated with transcriptional regulation of the cassette.

**Figure 3:**
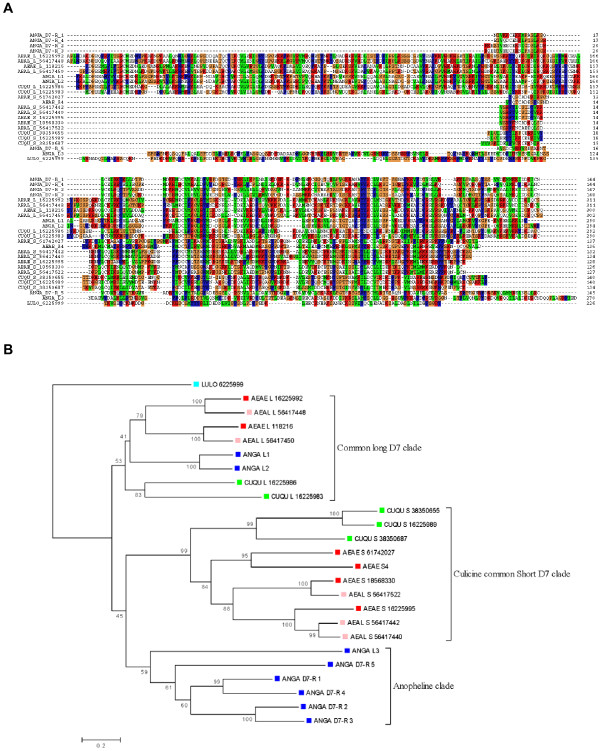
**Comparison of the D7 salivary proteins from *Ædes ægypti *(AEAE), *Æ. albopictus *(AEAL), *Culex quinquefasciatus *(CUQU), *Anopheles gambiæ *(ANGA), and *Lutzomyia longipalpis *(LULO)**. **A**, Clustal alignment. **B**, Phylogram showing the bootstrap values.

Alignment of the D7 sequences from *Æ. ægypti *with those of *Æ. albopictus, Culex quinquefasciatus, An. gambiæ*, and one D7 salivary protein from the sand fly *Lutzomyia longipalpis *indicates, as shown before, that the short D7 proteins appear to be truncated versions of the long D7 proteins, which appear to be the ancestral type (Figure 3A). The resulting phylogram, using the sand fly sequence as an outgroup, shows three clades without strong bootstrap support; however, the inner tree branches show considerably more conservation of the multiple forms within genus than between genera. For example, the common long D7 clade (Figure 3B) shows that two of the long D7 proteins of *An. gambiæ *are more closely related to each other as are the Culex or the Ædes pair. Within the Ædes genus, the *Æ. albopictus *and *Æ. ægypti *homologues are distinctly grouped together, indicating that they share a relatively recent common ancestor before the duplication event, as expected from these two mosquitoes of the same subgenus. The same pattern is visible in the culicine short D7 clade (Figure 3B) where all short D7 proteins are more related to each other within genus than between genera. This is even more remarkable in the short D7 proteins of Anopheles, where all short D7 proteins form a single clade outside of their culicine counterparts. If the gene duplication events that lead to the formation of long and short D7 proteins occurred in the primordial mosquito ancestral to both culicines and anophelines, the tree pattern observed would be one where the orthologous pairs would be more similar to each other between genera than within genus. Two possible explanations may account for the observed tree pattern: either the gene duplications leading to the D7 expansions occurred independently after the division of the culicine and anopheline lineages, or some degree of gene conversion occurred within each species, maintaining the uniformity of the genes within species. This latter scenario is consistent with the proposed primordial role of the D7 proteins, e.g. sequestration of host serotonin released by thrombocytes at the site of bite, a function that would require the D7 proteins to be a major salivary protein constituent [3]. The gene duplication event would be beneficial in allowing increased transcript mass needed to create the substantial amount of protein needed in the mosquito saliva to chelate the near-micromolar concentration of the vasoactive amine. Gene conversion events to maintain this function on multiple genes could be beneficial at this earlier stage of blood feeding evolution. This phenomenon would maintain intraspecific copies of the gene family more similar to each other than to the orthologous interspecific copies. With time, other salivary proteins may have taken a similar role of preventing platelet function, allowing the D7 proteins to acquire different functions such as binding other amines or to become anti-bradykinins, a function apparently only acquired in the anophelines, which diverged from the culicines ~150 million years ago [22]. For a review on the evolution of gene families, see references [24,25].

**Figure 4:**
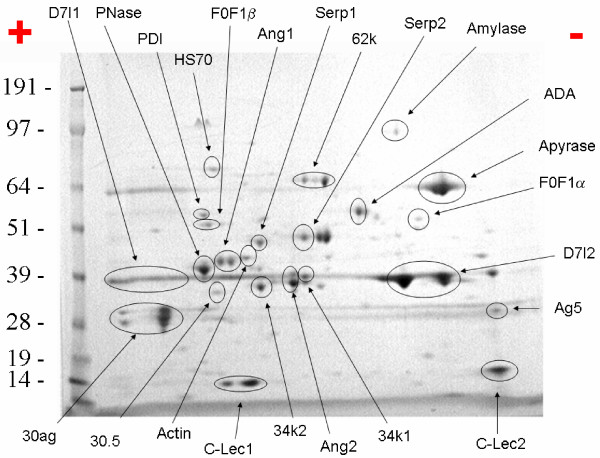
**Two-dimensional gel electrophoresis of 50 μg salivary protein from adult female *Ædes ægypti *mosquitoes**. Numbers on the left indicate molecular weight marker positions in the gel. The + and - signs indicate the anode or cathode side of the isoelectrophocusing dimension, which ranged from pH3-10. Gel bands that were identified to a protein (following tryptic digestion and mass spectrometry) are shown in the gel. In some cases, more than one band accounted for the same protein, possibly due to trailing or multiple isoforms. The proteins found were: D7l1 (gi: 16225992), PNase (purine nucleosidase, gi: 21654712) PDI (protein disulfide isomerase, gi: 94468800), HS70 (heat-shock protein 70 KDa, gi: 94468818), F0F1β (F0F1 ATPase beta subunit, gi: 94468834), Ang1 (angiopoietin-like protein, gi: 18568298), Serp1 (salivary serpin1, gi: 18568304), 62 k (62-kDa proteins gi: 18568300 and gi: 18568302), Serp2 (salivary serpin2, gi: 94469320), amylase (gi: 2190949), ADA (adenosine deaminase, gi: 18568326), apyrase (gi: 1703351), F0F1α (F0F1 ATPase, alpha subunit, gi: 94468442), D7l2 (gi: 118216), Ag5 (antigen5 protein, gi: 18568284), C-Lec2 (C-type lectin, gi: 94468370), 34k1 (34-kDa protein 1, gi: 94468642), Ang2 (angiopoietin-like protein2, gi: 94468352), 34 k2 (34-kDa protein2, gi: 18568296), C-Lec1 (C-type lectin 1, gi: 18568318), actin (gi: 94468486), 30.5 (30.5-kDa salivary protein, gi: 61742033), and 30ag (30-kDa antigen, gi: 18568322). For experimental details, see Methods.

**Figure 5:**
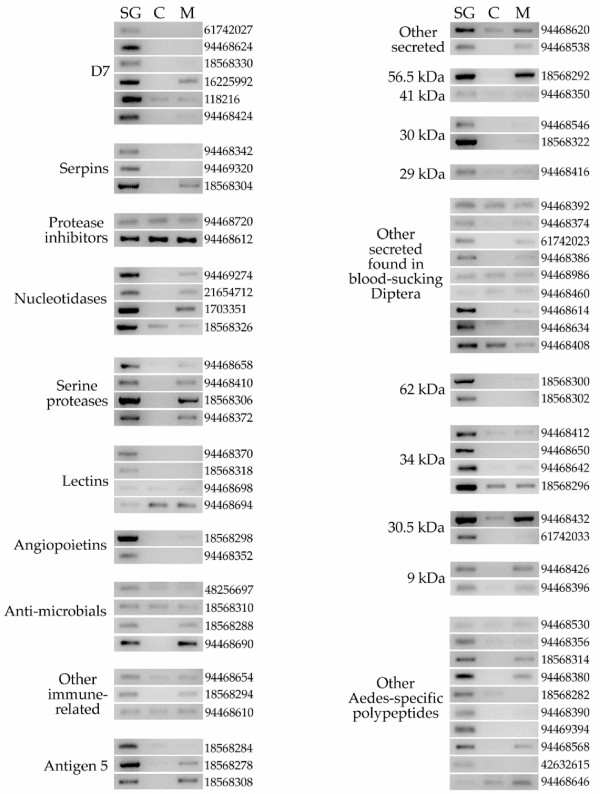
**Tissue and gender expression specificity of salivary gland genes of *Ædes ægypti***. RT-PCR results determining gene expression in female salivary glands (SG), carcass of female mosquitoes (C), and whole male mosquitoes (M) for selected genes in the sialotranscriptome of *Ædes ægypti*.

Evidence for synthesis of the two large D7 proteins and for D7s2 was shown before from Edman degradation results of SDS-PAGE gels [3]. Presently, we observed extensive coverage of tryptic fragments for both long D7 proteins (D7l1 and D7l2) as shown by two-dimensional (2D) gel electrophoresis (Figure 4 and Additional File 2) [14]. This protein family appears polymorphic. The predicted translation products of some of these alleles are shown in Additional File 2. As expected from this protein family, all transcripts described in Additional File 2 are more expressed in the SG cDNA libraries than in the remaining libraries, three of which are significant by the χ^2 ^test at the 0.05 level. RT-PCR experiments agree with these results, indicating a selective or preferential expression of this gene family in female SG (Figure 5 and Table 2). It should be noted, however, that transcripts encoding the short D7 were exclusively found in female glands, whereas mRNA encoding the long D7 were also detectable at a lower level in adult males (D7l1, D7l2) and in other female tissues (D7l2). This observation may be connected to an independent regulation of the short and long D7 cassettes, which are more that 30 kb apart.

###### Phosphatidylethanolamine binding proteins

We report three members (one possibly truncated) of this very ubiquitous protein family never found before in sialotranscriptomes of blood-sucking arthropods [26,27], two of which have clear signal peptide indicative of secretion. This protein family is known to bind lipids and was also shown to have serine protease inhibitory capacity. Their role in saliva is unknown. No enrichment was found for these transcripts on the SG libraries when compared with other libraries.

###### Other small molecule binding proteins

An OBP and a lipocalin with a juvenile hormone binding motif were found in the sialotranscriptome of *Æ. ægypti*, both containing distinct peptide signal indicative of secretion. The D7 proteins belong to the OBP superfamily. Lipocalins are abundantly expressed in tick and triatomine sialomes, where they act as nucleotide (nt)-and biogenic amine-binding proteins in addition to other functions. Their function in Ædes is unknown. No enrichment was found for these transcripts on the SG libraries when compared with other libraries.

##### Secreted protease inhibitors

###### Serpins

Two serpins have been described before in *Æ. ægypti*, one of which has been characterized as an inhibitor of FactorXa of the clotting cascade [28,29]. We present one allele of the FXa-directed anticoagulant precursor having only 89% identity to the reported protein[30] which originated from the Rockefeller strain of *Æ. ægypti*, and two alleles of a novel salivary serpin mapping to supercontig1.65. The three genes coding for these serpins are not located near each other in the *Æ. ægypti *genome. All three salivary *Æ. ægypti *serpins have corresponding homologues found in *Ae. albopictus *sialotranscriptome[31]. The novel serpin has abundant tryptic fragment matches recovered by proteomics (band marked Serp2, Figure 4), indicating its expression in the SG, as did gi|18568304 (marked Serp1, Figure 4). Transcripts for all serpins are significantly overrepresented in the SG library when compared with the remaining libraries, in accordance to the RT-PCR experiments shown in Table 2, which indicates that two of the three serpins are female specific and that one may be found also in males but not in carcasses of females not containing SG (Figure 5).

###### Other protease inhibitors

A Kazal domain-containing peptide, similar to one described in *Ae. albopictus* and to several other proteins described as thrombin inhibitors, was found in the *Æ. ægypti *sialotranscriptome. A cystatin was also found, but this protein is reported as truncated; we were not successful in searching the genome for the missing exon(s). This could be due to the large intron size observed in *Ae. aegypti*. Accordingly, no indication of secretion is possible, but it is described in this section due to the importance of this family in inhibiting proteases associated with inflammation. Both transcripts are ubiquitously found in mosquito tissues by RT-PCR and may play a housekeeping role.

##### Vasodilator

###### Sialokinin

The gene coding for this endothelium-dependent peptide vasodilator [32,33] has been reported earlier and shown to be transcribed specifically in female SG [34]. Although two forms of the peptide have been described earlier differing in the aminoterminal (aspartate or asparagine), only one gene is found coding for this peptide sequence, for which 60 EST were found in the salivary cDNA library. The Asn version may have been an artifact from the modification of the original peptide, which was stored in acidic solution.

##### Enzymes

###### Nucleotidases

**Figure 6:**
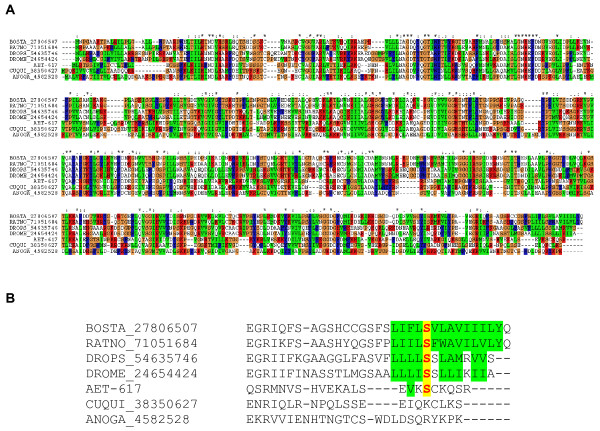
**Alignment of members of the 5' nucleotidase family deriving from salivary glands of mosquitoes or from *Drosophila melanogaster, D. pseudoobscura, Bos taurus*, or *Rattus rattus***. **A**, Total aligment. **B**, Alignment on the carboxyterminal region. The numbers in the sequence titles indicate the NCBI accession number. Notice the conserved serine (where the inositol phosphate membrane anchor binds) surrounded by hydrophobic amino acids in the nonsalivary enzymes.

The salivary purinergic degradation machinery of *Æ. ægypti *comprises the enzymes apyrase (a member of the 5' nucleotidase family), adenosine deaminase (ADA), and purine hydrolase [35-37], which may serve an antihemostatic and antiinflammatory function by removing nucleotide agonists of platelet aggregation and mast cell degranulation. In addition to these previously described enzymes, we found a second 5' nucleotidase that may function either as an alternative apyrase or as a secreted salivary 5' nucleotidase, as is the case with *Lutzomyia longipalpis *[38]. The novel 5' nucleotidase has only 38% identity to the previously characterized apyrase form of *Aedes aegypti*[39] but has a higher identity (52%) to a *Culex. quinquefasciatus *salivary 5' -nucleotidase/apyrase protein[40]. 5' nucleotidases are typically seen in the external part of the cellular membrane to which they are bound by a inositol phosphate anchor [41-43]. Secreted apyrases and 5' nucleotidases have lost either the conserved Ser or the surrounding lipophylic amino acids (aa) (or both) to which the inositol phosphate moiety binds to [35,38]. The novel Ædes 5' nucleotidase, like the previously described salivary apyrase [35], lacks the typical Ser residue surrounded by hydrophobic aa typical of membrane-bound enzymes, similarly to other mosquito salivary 5' nucleotidase (Figure 6), supporting their role as secreted 5' nucleotidases. This novel apyrase may contribute to the purinergic degradation machinery found in saliva of *Æ. ægypti*. All these genes are overrepresented in SG libraries and, except for ADA, significantly so. RT-PCR results are somewhat contradictory with the proposed role of these enzymes in blood feeding: with the exception of the ADA coding transcript that was enriched in female SG, the other genes appeared to be SG specific, because they are expressed in SG of females and in whole males, which would suggest a role in sugar feeding, instead. We do not have a good explanation for this observation; however, we should point out that apyrase, purine nucleosidase (PNase), and ADA showed very similar expression profiles by RT-PCR in the related mosquito *Æ. albopictus *(Arcà *et al*., manuscript in preparation). Evidence of synthesis of these enzymes was found for the ADA, the original apyrase, and the PNase, which provided abundant tryptic fragments (Figure 4, bands labeled ADA, apyrase, and PNase).

A novel ribonuclease of the T2 family[44] was also characterized. This enzyme has not been previously characterized in sialotranscriptomes. It has a typical signal peptide indicative of secretion and may function in the degradation of extracellular RNA [45].

###### Serine proteases

Nine secreted serine proteases varying in predicted mature molecular weight between 28 and 43 kDa were found in the *Æ. ægypti *sialotranscriptome, seven of which are being reported for the first time (Additional File 2). Two of these serine proteases (AEA-876[46]and AEA-562[47]) contain a CUB domain[48], indicating specialized substrate recognition. Both are found in supercontig1.217 within 63 kb of each other. Some of these enzymes (such as gi|18568334[49]) are possibly related to immunity and are similar to other enzymes annotated as prophenoloxidase (PPO)activators, but they could have been co-opted to function in hydrolyzing specific host proteins. A smaller number of this type of enzymes found in *An. gambiæ *sialotranscriptomes was selectively expressed in the SG of adult females, indicating they may play a role in blood feeding, perhaps by activating antiinflammatory pathways (such as protein C) or deactivating inflammation. One such Ædes enzyme is AE-226[50], similar to proteins annotated as chymotrypsin, which is overexpressed in Ædes sialotranscriptomes as opposed to the remaining transcriptomes. Three of these serine proteases were significantly underrepresented in the nonsalivary-gland libraries. Four serine proteases tested by RT-PCR agreed with the library frequency results: the transcripts were found in female glands and adult males but not in female carcasses deprived of SG.

###### Sugar hydrolases

Previously reported amylase[51,52] and alpha-glycosidase/maltase[53,54] are abundantly overrepresented in the salivary EST collection. These genes were shown to be expressed in the proximal regions of the female glands, the region associated with sugar feeding.

###### Other hydrolases

An alkaline phosphatase[55] and a carboxylesterase[56], both containing signal peptides indicative of secretion, are described. Both enzymatic activities in adult female SG have been previously described in Ædes [57], and the esterase activity shown in saliva, but their function in blood feeding can only be speculated upon.

##### Immunity-related proteins

The SG of mosquitoes produce various antimicrobial polypeptides and other immunity-related products such as bacterial surface-recognizing proteins and lectins that may be important in opsonization and initiation of activation of the PPO enzyme leading to pathogen melanization. The purpose of these products may be to control microbial growth in the sugar solutions stored in the crop or in the gut following a blood meal. Previously, lysozyme[58] was found in both male and female SG of *Æ. ægypti*, and in the mosquito crop [59] and shown to be secreted by females following either a sugar or blood meal [60]. Indeed, *Æ. ægypti *salivary lysozyme is significantly overrepresented in the SG libraries, and the RT-PCR experiment supports salivary expression in both male and female SG (Table 2 and Figure 5). Anopheline mosquitoes also display a similar pattern of lysozyme expression in the proximal gland region [61]. It is possible that most immunity-related gene products follow the same pattern of expression shown by lysozyme. Some of the enzymes possibly associated with PPO activation have been listed above in the Enzyme section.

###### C-type lectins

Five C-type lectins are described in Additional File 2, four of which are novel. Expression of AE-189 and gi|18568318 was confirmed by mass spectrometry following tryptic digestion of protein bands (labeled C-Lec1 and C-Lec2 in Figure 4). These two lectins are also expressed significantly more in sialotranscriptomes than in other *Æ. ægypti *cDNA libraries, indicating that they are possibly salivary-tissue specific. In accordance with this expectation, RT-PCR experiments indicate expression exclusively in female SG (Table 2 and Figure 5). The two genes coding for these salivary-specific lectins were found as an inverted tandem repeat in supercontig1.10, each with a single intron separating the signal peptide gene region from the remaining coding sequence. Two other C-type lectins tested by RT-PCR were ubiquitously expressed.

The C-type lectin family is expressed in most mosquito sialotranscriptomes described thus far. This protein family is implicated in immune recognition phenomena in general and in Plasmodium development in Anopheles in particular [62,63]. Despite these not-yet-demonstrated roles of C-type lectins in salivary immunity, it is interesting that in snake venoms this protein family has been recruited to perform various unrelated functions such as anticlotting, toxin, and platelet aggregation inducer [64,65]. Lectins may also play a role in the colectin pathway of complement activation [66]. Hemagglutinins were described in anopheline SG more than 60 years ago [67]. This activity may help concentration of red blood cells in the mosquito gut [68]. The molecular nature of any anopheline hemagglutinin, however, is unknown. Differently from anophelines, and despite having salivary lectins, Ædes SG homogenates lack hemagglutinins, indicating that the salivary lectins do not recognize vertebrate red blood cells or that they are monomeric in their carbohydrate binding site. Overall, it appears that the two female SG-specific lectins may have a role in hemostasis rather than immunity.

###### Other proteins with sugar-binding domains

AET-12005 and AET-670 are similar to, but shorter than, N-acetylgalactosaminyltransferase and glucuronyltransferase, respectively, appearing to derive from novel genes that arose from gene duplications and partial deletions of ancestral genes coding for carbohydrate binding enzymes, the final products lacking the original carboxyterminal domain. AET-12005 has a partial Pfam Glycos_transf_2 motif that comprises a diverse family transferring sugar from UDP-glucose, UDP-N-acetyl-galactosamine, GDP-mannose, or CDP-abequose to a range of substrates including cellulose, dolichol phosphate, and teichoic acids. AET-670 has a weak match to the PFAM UDPGT motif and is similar to proteins in the nonredundant (NR) database annotated as UDP-glucosyl transferase. It is possible that these proteins have a destination in the endoplasmic reticulum or Golgi and do not have a secretory nature. Their function is unknown.

The proteins annotated as imaginal disk growth factor protein 4[69] and AEA-871BRE were expressed in the sialotranscriptome of *Æ. ægypti*. These proteins have a chitinase domain and are homologous to *An. gambiæ *bacteria responsive protein 1[70] and bacteria responsive protein 2[71], which were shown to be immune-responsive chitinase-like proteins that have lost chitin-binding activity [72].

###### Angiopoietins/ficolins

This group of proteins has the PFAM fibrinogen C motif[73]seen in invertebrate proteins displaying lectin activity toward N-acetylglucosamine residues and implicated in immune function [74]. In *An. gambiæ*, the ficolin family was expanded in comparison to *Drosophila melanogaster*, where 53 members were seen in its genome as opposed to 20 in the fruit fly [75]. Three proteins belonging to this family are shown in Additional File 2, two of which are novel. Evidence for salivary expression of gi|18568298 and AE-154 was found by mass spectrometry in tryptic digests of protein bands (labeled Ang1 and Ang2, respectively, in Figure 4). Of interest, the two genes for these proteins occur as a tandem repeat in supercontig1.15. EST for these two genes are also overrepresented in the sialotranscriptomes and are indicated to be female-salivary-gland specific by RT-PCR experiments, thus suggesting a blood-feeding rather than an immune role for these proteins.

###### Antimicrobial peptides (AMP)

The gene products for the AMP gambicin[76], lysozyme[58], and defensin A1[77] have been previously described in *Æ. ægypti *sialotranscriptomes and are listed in Additional File 2. Transcripts encoding gambicin and defensin A1 were detected by RT-PCR in all tissues examined, indicating ubiquitous expression (Table 2 and Figure 5); however, a significant overrepresentation of the corresponding EST in sialotranscriptomes should be pointed out. We additionally describe three novel peptides that may have an antimicrobial function. AET-590[78] has GY repeats that are also found in peptides of similar size known to have antimicrobial activity in nematodes [79]. AET-462[80] and AET-11358[81] are candidate AMP containing a HHH motif seen in other histidine-rich AMP [82,83]. AET-11358 appears to be SG specific, as a total of 88 EST was found in the combined SG transcriptome, although none were seen in other tissue transcriptomes. RT-PCR confirmed the presence of the transcript in female glands and male bodies but not in female carcasses without SG (Table 2 and Figure 5).

###### Other immune-related gene products

A peptide (named AEA-233[84]) closely related to a previously described *Æ. ægypti *peptide named i23R[85] potentially involved in Plasmodium susceptibility [86] was found in the sialotranscriptome. We also present an allele to AEA-233a, indicating the polymorphism of this gene. The *Æ. albopictus *sialotranscriptome revealed a homologue that is 63% identical, but no significant matches were found to any other animal or plant proteins in the NR database. This peptide may belong to a not-yet-characterized antimicrobial gene family specific to the Ædes genus. Expression of AEA-233a was ubiquitous by RT-PCR.

Two other gene products are described, both associated with pathogen surface-pattern recognition: the previously described Gram negative binding protein[87], which is significantly overrepresented in sialotranscriptomes and appears expressed both in female SG and in adult males (Table 2, Figure 5), and the novel AE-7210[88], which is similar to peptidoglycan recognition proteins and was ubiquitously expressed by RT-PCR experiments.

###### Mucins

Mucins and peritrophins are proteins associated with lining of epithelia or inert extracellular structures, such as chitin. Mucins are highly glycosylated proteins containing Ser or Thr modified with N-acetylgalactosamine residues. Their expression in the SG may have a function of lining the chitin surfaces of the mouthparts, but they may also assist in antimicrobial functions.

We present 12 mucins in Additional File 2, 11 of which are novel, including one allele. These proteins have an average Ser+Thr equal to 13.8% of their total aa, as opposed to 0.9% observed as the average of all proteins found in Additional File 2. We additionally report on a polypeptide (AE-466, mucin-like peritrophin) containing three glycosylation sites and one chitin-binding domain, which may be involved in proximal lining of the cuticular duct. All other proteins have 11–69 glycosylation sites.

#### Putative secreted proteins without functional classification

##### Belonging to ubiquitous protein families

###### Antigen5 (AG5) family

AG5-related salivary products are members of a group of secreted proteins that belong to the CAP family (cysteine-rich secretory proteins; AG5 proteins of insects; pathogenesis-related protein 1 of plants) [89]. Members of this protein family are found in the SG of many blood-sucking insects [3,90,91]. Most of these animal proteins have no known function; in the few instances to the contrary, they diverge from proteolytic activity in Conus [92], to smooth muscle-relaxing activity [93,94] in snake venoms, to salivary neurotoxin in the venomous lizard *Heloderma horridum *[95]. Three members of this gene family were previously described in the sialotranscriptome of *Æ. ægypti*. EST's for all three genes are overrepresented in the sialotranscriptome as compared with the combined transcriptomes, indicating they may be preferentially expressed in the SG. In accordance with these results, gi|18568284 was exclusively transcribed in female glands as indicated by RT-PCR, suggesting an antihemostatic function for the gene product, while the other two genes are transcribed in female glands and male bodies but not in female carcasses.

Differently to *An. gambiæ*, which has four salivary AG5 members, three of which cluster in chromosome arm 2 L [12], the salivary AG5 proteins of Ædes do not appear to cluster in the genome, mapping to different supercontigs.

#### Other secreted proteins of unknown function found in non-bloodsucking insects

Eight putative secreted proteins have similarities to proteins or protein domains found in non-bloodsucking insects. One of these proteins (AE-796) [96] is a truncated fragment where it is not possible to identify whether it has a signal peptide indicative of secretion, but it has a weak CDD domain PAN_AP_HGF[97], which is found in plasminogen/hepatocyte growth factor proteins, and various proteins found in Bilateria, such as leech antiplatelet proteins; however, the mRNA encoding this protein was found ubiquitously expressed by RT-PCR and may not have a unique salivary role in blood feeding. AE-389[98] has a TIL[99] domain (trypsin inhibitor like) and is significantly overexpressed in sialotranscriptomes. RT-PCR indicates both male and female SG may be the target tissue of expression of this peptide (Figure 5 and Table 2). Peptides containing a TIL domain were also found in the *An. stephensi*[100] and *An. gambiae *adult male sialotranscriptomes[101-103]. The finding of this type of peptide being overexpressed in male *An. gambiæ *SG indicated a possible antimicrobial function rather than a function as a host serine protease inhibitor during blood feeding. Indeed, a tick TIL domain containing peptide named ixodidin[104] was found to have an antimicrobial function in addition to inhibiting serine proteases [105]. The remaining six polypeptides have similarities to Drosophila or other species, and their structure does not hint at any particular function.

##### Belonging to families only found in blood-sucking diptera

###### 56-kDa family

This protein family has been found to date only in salivary transcriptomes of adult mosquitoes, including adult male *An. gambiæ*. The SG specificity of this gene transcript in *Æ. ægypti *is supported by significant overrepresentation of EST on the sialotranscriptome and by RT-PCR (Figure 5 and Table 2). All family members have a signal peptide indicative of secretion and a predicted molecular weight near 56 kDa. BLAST comparisons[106] also show weak similarity to bacterial proteins but to no other eukaryotes. Following 4 iterations of PSI-BLAST[107], only mosquito and bacterial proteins are retrieved [108], suggesting that this family of proteins may have originated as a lateral transfer from a bacterial genome to the ancestral mosquito genome. The single exon structure of the gene[109] – unusual in eukaryotes, particularly for a protein of this size, but the rule in prokaryotes – supports this hypothesis. The *An. gambiæ *homologue also displays a single exon gene structure [110], as reported previously [12]. The bacterial proteins retrieved by PSI-BLAST are mostly annotated as phage-associated proteins, suggesting the lateral transfer might have occurred via a phage-associated mechanism.

###### 41-kDa family

Two novel alleles coding for this protein family are described in Additional File 2. The gene coding for this protein has a 3 exon structure[111] on supercontig1.116. The gene product shows similarities to proteins of comparable size found in *Æ. albopictus *and *Culex pipiens *and to a shorter protein in salivary transcriptomes of *Culicoides sonorensis*. A match to *Æ. ægypti *gi|61742035[112] probably represents a misannotated protein with a DNA frame-shift error. Three iterations of PSI-BLAST[113] only retrieved culicine and Culicoides proteins, indicating the uniqueness of this protein family. No salivary anopheline sequences similar to the 41-kDa family have been reported, including all ENSEMBL-predicted *An. gambiæ *proteins deposited at the National Center for Biotechnology Information (NCBI) (which contains all proteome versions released by ENSEMBL). EST coding for the two alleles in *Æ. ægypti *are significantly underrepresented in the non-SG libraries; RT-PCR suggests ubiquitous expression of one of the alleles, although only very faint bands were detected even after 35 cycles of amplification (Figure 5). The function of any member of this protein family is unknown.

###### 30-kDa GE-rich family

This acidic, Gly/Glu-rich protein family is abundantly expressed in adult female mosquito SG, where they appear to be involved in allergic reactions to mosquito bites [114]. In *Æ. ægypti*, two proteins of this family have been previously reported, and we now report two additional splice variants and alleles. Evidence for expression was found in bands labeled 30 ag (for gi|14423642[115] and gi|18568322[116]) in the 2D gel experiment shown in Figure 4. The two proteins are coded by an inverted tandem repeat in supercontig1.464 separated by only 363 base pairs. The sialotranscriptome is significantly overrepresented in EST coding for this protein family, indicating it is salivary specific. RT-PCR confirms the female SG specificity of these transcripts. Based on the public sequences available, it appears that in anopheline mosquitoes (*An. gambiæ, An. stephensi, An. albimanus, An. dirus*), only one polymorphic gene exists for this protein family per genome.

###### 29-kDa family

**Figure 7:**
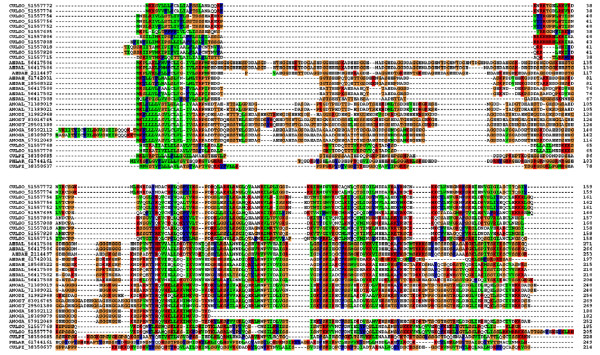
**Clustal alignment of the 29/30-kDa protein family in hematophagous Diptera**. The letters represent the species *Ædes ægypti, Æ. albopictus, Culex pipiens, Culicoides sonorensis, Phlebotomus ariasi, Anopheles albimanus, An. gambiæ, An. stephensi*, and *An. dirus *using the three first letters of the genus and two letters of the species name. The numbers are NCBI accession numbers.

**Figure 8:**
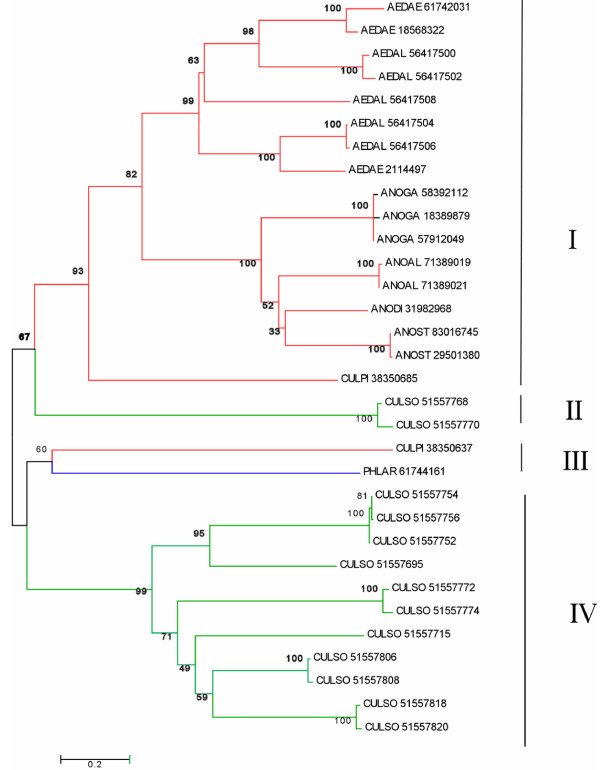
**Phylogenetic tree of the 29/30-kDa protein family in hematophagous Diptera**. The numbers in the tree nodes indicate the bootstrap values. Tree branches in red represent mosquito species; in blue, sand fly; and in green, Culicoides. The bar at the bottom of **B **indicates amino acid divergence between sequences. See also legend of figure 7. The bar at the bottom of **B **indicates amino acid divergence between sequences.

Two different transcripts[117] in *Æ. ægypti *are possibly obtained from the same genomic region coding for the basic (pI = 9.4) salivary protein AE-236 and for the alternative shorter transcript AE-236A, which was not found on the salivary EST but rather as one contig assembled from four EST deriving from nonsalivary libraries[118]. BLAST comparison of the deducted salivary protein with the NR database shows similarities to other culicine and anopheline salivary proteins, including weak similarities to some members of the 30 kDa protein family. Four iterations of Psi-blasT[119] are able to assemble only salivary proteins of mosquitoes, Culicoides, and Phlebotomus, including all 30-kDa proteins discussed above, suggesting that either this unique protein family was co-opted as salivary proteins independently by these different families of Diptera or that they have a common blood-feeding ancestor. ClustalW alignment of the sequences shows that following the signal peptide region, a subset of the proteins have a Ser/Thr/Gly-rich region, poor in aliphatic aa as shown in Figure 7 by the richness in brown residues. The carboxyterminal region is marked by the alternation of polar and aliphatic residues. A subset of Culicoides proteins does not have this domain. The phylogenetic tree shows a robust mosquito clade (marked I in Figure 8) with three members of the family per Ædes species (two 30-kDa genes, one 29-kDa gene, plus alleles) and one member (plus alleles) per Anopheline species, which have only a single 30-kDa member. A single *Cx. pipiens *sequence is also part of this clade. CladeII has two very similar Culicoides proteins. CladeIII has very divergent Culex and Phlebotomus sequences, and CladeIV has solely Culicoides sequences, representing those lacking the Ser/Thr/Gly-rich aminoterminal domain. It is tempting to speculate that these data support a common origin of blood feeding for these three Dipteran families, where two genes are found in Culicines, a single in Anophelines and possibly Phlebotomus, and a rather large gene expansion in Culicoides, which has at least seven genes in the family. Notice that CladeIII, containing mosquito and sand fly sequences, roots with Culicoides CladeIV and that Culicoides cladeII roots with the mosquito cladeI, indicating that Culicoides may have shared a common ancestor with mosquitoes that had two genes of this unique family. Alternatively, convergent evolution may have shaped these genes to produce similar proteins. AE-236A was enriched in the SG of adult females, while AE-236 was significantly underrepresented in non-SG libraries, suggesting a salivary specificity for this protein family, as is the case with the related 30-kDa family discussed above.

###### Other mosquito-or Diptera-specific peptides

Additional File 2 also includes 14 additional polypeptides, one of which is an allele, showing sequence similarities to putative proteins from other hematophagous Diptera including, in a few cases, some weak similarities to Drosophila; 13 of these are novel. Among this class of polypeptides, nine were analyzed by RT-PCR (Figure 5 and Table 2), and seven are significantly underrepresented in the non-sialotranscriptomes, as follows: AE-212[120], which is similar to Drosophila and Culicoides proteins of unknown function and was ubiquitously expressed by RT-PCR experiments; two alleles (AE-165[121] and AE-163[122]) coding for basic (pI = 9.8) 29-kDa proteins containing 4 putative galactosylated Ser/Thr and similar to Culex and Culicoides[123] salivary proteins; three polypeptides whose expression appeared gland specific, as suggested by RT-PCR (AE-196, which is similar to the *An. gambiae *gSG8[124] salivary protein; gi|61742023, which is similar to tryptophan-rich salivary proteins of Culex[125]; and AE-209, similar to another Culex salivary protein[126]). AE-225 (ubiquitously expressed by RT-PCR experiments), is weakly similar to proteins varying in size from 150–180aa residues from Anopheles[127] and Drosophila[128]. Two additional transcripts, AE-937 and AE-752, were expressed solely in the adult female SG by RT-PCR. The function of these proteins remains to be investigated.

##### Genes belonging to protein families found to date only in Ædes genus

Nineteen genes were found expressed in the sialotranscriptome of *Æ. ægypti *coding for polypeptide families known only in the Ædes genus, as follows:

###### 62-kDa family

Two single exon[129] genes separated by ~20 kb in supercontig1.15 code for proteins with signal peptides and mature mass of 62–63 kDa. Transcripts for these genes are significantly overrepresented in the sialotranscriptome and are shown to be adult female SG specific by RT-PCR (Figure 5 and Table 2). They are similar to homologous salivary protein sequences seen in *Ae. albopictus*[130] and, to a much smaller degree, to rhoptry proteins of Plasmodium. Repeated Leu and Glu residues provide similarities to myosin[131], indicating this protein family may be involved in adhesion phenomena. Their uniqueness among metazoan and single-exon structure indicates possible horizontal acquisition of this gene family in Ædes. Both genes are abundantly expressed in the SG as evidenced by bands labeled 62 k by 2D gel electrophoresis MS/MS (Figure 4).

###### 34-kDa gene family

**Figure 9:**
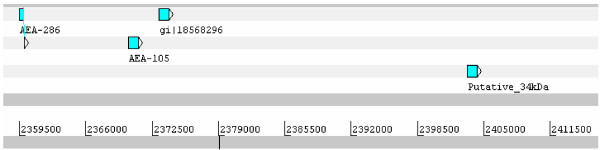
The 34-kDa gene region in supercontig1.92.

Seven transcripts coding for related proteins were found mapping to supercontig1.92. After locating the corresponding genomic regions, these 7 transcripts were annotated as truncated forms or alleles of three genes found as a tandem repeat (Figure 9). We additionally found one possible related gene in the most distal region of the 34-kDa cassette (Putative_34 kDa in Figure 9). Except for the first gene on the cassette, which codes for a 16-kDa protein and has two exons[132], the remaining genes are single exonic[133] and code for proteins of ~34 kDa. The two central genes, each with a single exon, are abundantly expressed as evidenced by MS/MS sequencing of tryptic digested bands (34k1 and 34k2 in Figure 4). All transcripts matching this gene region are significantly overexpressed in the SG transcriptome when compared with the remaining libraries. RT-PCR indicates they are enriched or exclusive of adult female SG (Figure 5). Protein products of these genes match significantly only *Ae. albopictus *proteins[134]. PSI-BLAST for each transcript against the NR protein database reveals cytoskeletal proteins such as actin and myosin, mainly due to the presence of repeated charged aa. This indicates that this protein family may be associated with adhesion phenomena (not shown). The single-exon nature of most members of this gene family and their uniqueness among Metazoa points to a horizontal acquisition of this gene.

###### 30.5-kDa family

Two genes coding for proteins of ~30.5-kDa (not to be confused with the 30 kDa/GE-rich protein family) are found as a tandem repeat on supercontig1.280. The gene coding for gi|61742033 is abundantly expressed as evidenced by MS/MS of tryptic digested band labeled 30.5 in Figure 4. These proteins are similar only to homologues[135] found in *Ae. albopictus*[136]. Both genes are significantly overtranscribed in the sialotranscriptome when compared with other transcriptomes. RT-PCR indicates enrichment in the female SG or exclusive expression in the same organ.

###### 9-kDa family

Two genes[137] having 80% sequence similarity and coding for mature peptides of 8.5 and 9.5 kDa are found as a tandem repeat in supercontig1.18. They are similar only to salivary peptides of *Ae. albopictus*[138]. Both genes are significantly overexpressed in sialotranscriptomes. RT-PCR suggests that these genes occur in both male and female SG.

###### Other salivary polypeptides

Additional File 2 lists an additional 11 full-length transcripts originating from 10 genes coding for proteins found to date only in the genus Ædes. Six of these genes have overrepresentation in the sialotranscriptome, as follows: AE-376[139], AE-156[140], gi|18568314[141], gi|18568282[142], AE-211[143], and AE-214[144]. RT-PCR in 10 of the 11 transcripts show enrichment or female specificity for 5 transcripts and ubiquitous expression for 2 genes, while 3 appear to be transcribed in both male and female SG (Figure 5, Table 2, and Additional File 2). Their function is unknown.

### Proteins of possible housekeeping function

#### Function possibly predicted

##### Transporter function and storage proteins

Being a secretory organ, mosquito SG are involved in active ion and water transport associated with their function. V-ATPases are generic 'batteries' that generate a proton gradient across membranes that can be coupled with ion exchangers and are used in eukaryotic cells for transport purposes [145]. This multi-subunit enzyme complex has been extensively studied in insects, in particular in Lepidotera larvæ midgut [146] and in the malpighian fluid transport in mosquitoes [147]. A role for V-ATPases in mosquito SG secretion has been previously proposed [148]. Additional File 2 reports 22 gene products including 9 subunits of the V-ATPase complex, 3 aquaporins (water channels), 2 chloride channels, and the enzyme carbonic anhydrase, which is associated with proton transport in epithelia to speed intracellular pH regulation via the CO_2_+H_2_O↔bicarbonate + H^+ ^reaction.

##### Probable signal transduction function

Thirty-eight proteins, 35 of which are novel, are described in Additional File 2 as possibly associated with signal transduction events. Included are four proteins associated with inhibition of apoptosis, four enzymes associated with juvenile hormone metabolism, one associated with ecdyesteroid metabolism, and a gamma-amino butyric (GABA) receptor-associated protein, in addition to protein kinases and phosphatases.

##### Nuclear regulation, transcription factors, and transcription machinery

Twelve (all novel) proteins are associated with nuclear function including histones, zinc finger proteins, and proteins associated with cell division. Additionally, we found 9 possible transcription factors and 21 proteins involved in the transcription machinery, only one of which has been previously reported (Additional File 2).

##### Ribosomal proteins and other proteins associated with the translation machinery

Being an organ involved in abundant protein synthesis, it is not surprising that many ribosomal and other translation associated proteins were identified. Additional File 2 lists 75 ribosomal proteins, 65 of which are novel for *Æ. ægypti*. Twelve of these ribosomal proteins, although also found in other transcriptomes, are significantly overexpressed in the sialotranscriptome. These are AE-155, AE-198A, AE-430, AE-411, AE-288, AE-200A, AET-3179, AET-2065, gi|78214568, gi|11762110, AET-3168, and AET-3303.

Eighteen proteins (14 novel) were possibly associated with the translation machinery, including elongation factors, tRNA synthases, and translation initiation factors. Elongation factors 2, 2 alpha, and 2 beta were significantly overexpressed in the SG.

##### Protein modification and protein export machinery

Forty-four proteins (43 novel) were possibly associated with the protein modification machinery including enzymes associated with proline isomerization, disulfide bridge formation, glycosylation, and several chaperones. Thirty-six proteins (35 novel) are possibly associated with the protein export machinery, including signal peptidase complex, endoplasmic reticulum, Golgi, and vacuole proteins. The putative cargo transport protein EMP24 is overexpressed in the sialotranscriptome, although also found in other nonsalivary libraries. Evidence for expression of a protein disulphide isomerase was found by MS/MS results of the tryptic digestion of band labeled PDI in Figure 4.

##### Oxidant metabolism

One peroxiredoxin, one thioredoxin, two superoxide dismutases, one cytochrome P-450 enzyme, and one truncated catalase are among ten proteins associated with oxidant metabolism (eight of which are novel). The cytochrome P-450 enzyme has a signal peptide indicative of secretion and is a member of the CYP4 family (based on the nomenclature of related proteins[149]), but it is included as a possible housekeeping function due to its high similarity to other insect enzymes[149] and because these enzymes normally need an associated reductase driven by NADPH+, which is normally only found intracellularly. Members of the CYP4 family can be found in peroxisomes, where they can be associated to arachidonic acid or eicosanoid reactions [150]. Of interest, AET-6749 is similar to a 40-kDa farnesylated protein associated with peroxisomes[151], indicating the presence of this organelle in Ædes SG and the possible reason for the signal peptide that may be needed for directing this enzyme to the peroxisome.

##### Proteasome and lysosomal machinery

Seventeen proteins (16 novel) were associated with the proteasome machinery, including several proteasome subunits and ubiquitin-related enzymes. Two previously described lysosomal enzymes are also listed in Additional File 2.

##### Cell metabolism

Forty-three proteins associated with nt, aa, carbohydrate, lipid, and heme metabolism or transport are described. AET-12468 is similar to enzymes annotated as kynurenine formamidase [152] and has a KOG motif indicative of this enzyme [153]; 9 transcripts were found among the 15,625 salivary EST but only 6 in the 217,296 nonsalivary EST, indicating this enzyme is overexpressed in the SG of Ædes. Kynurenine formamidase is a key enzyme in the degradation of tryptophan, producing L-kynurenine from N-formyl-kynurenine, the product of the action of L-tryptophan:oxygen 2,3-oxidoreductase on tryptophan, and a precursor to xanthurenic acid, which has been described in the SG of *An. stephensi *[154]. Xanthurenic acid has also been reported as the mosquito-derived gametocyte exflagellation factor of Plasmodium [155,156]. The presence of xanthurenic acid in Ædes saliva remains to be demonstrated, although a recent report indicates that Ædes mosquitoes deficient in the production of xanthurenic acid sustain normal *P. gallinaceum *development [157].

##### Energy metabolism

Fifty-four enzymes (51 novel) are presented as involved in energy metabolism. Most of these are mitochondrial constituents, a few of which are overrepresented in the salivary libraries compared with the other libraries, perhaps due to the larger-than average-salivary metabolism associated with protein synthesis and secretion. Evidence for expression of the alpha and beta subunits of the F0F1-type ATP synthase were found by MS/MS data obtained from bands labeled F0F1α and F0F1β on the 2D gel (Figure 4) indicating their abundant expression.

##### Cytoskeletal, adhesion, and extracellular matrix proteins

Fifteen proteins (14 are novel to Ædes) are associated with cytoskeletal, intercellular adhesion, or extracellular matrix functions, including actins, dynactin, tubulins and annexins, and the basal lamina protein named as SGS1, which was found to be a SG receptor for *P. gallinaceum *sporozoites [158]. Notably, this protein family has homologues only in *An. gambiæ*, where it was abundantly expressed in the SG of adult females [12] and in bacteria, the most closely related protein outside Anopheles being from the Wolbachia [12,158]. Four such large proteins (~200 kDa) were described in Anopheles, all intronless and contained in a tandem repeat on chromosome arm 3R, while six have been identified in Ædes, also intronless[159], and consistent with their horizontal acquisition from a Wolbachia bacteria. For more details on this protein family, see reference [158].

#### Probably housekeeping, function unknown

Fifty-nine proteins are described (all novel to Ædes) that are conserved with other organisms, thus characterizing the large group of 'conserved hypothetical' proteins [160] or are just hypothetical proteins with no similarities to other known proteins (five cases only). Two of the conserved hypothetical proteins are clearly membrane proteins of unknown function. Several members of this group are significantly overexpressed in the SG when EST's in the sialotranscriptome are compared with the remaining transcripts.

## Conclusion

Using high-throughput transcriptome analysis, we significantly expanded the *Æ. ægypti *SG transcript repertoire. A total of 614 transcripts was identified, 573 of which are new, and mostly full length. A subset of 136 transcripts was identified as possibly SG specific, 97 of which are novel. Analysis of tissue-specific transcription of selected genes revealed at least 31 genes whose expression is specific or enriched in female SG, whereas 24 additional genes were expressed in female SG and in males but not in other female tissues. Most of the 55 proteins coded by these transcripts have no known function and represent high-priority candidates for expression and functional analysis as antihemostatic or antimicrobial agents. This catalogue makes *Æ. ægypti *the mosquito vector for which the most complete salivary transcriptome is available. We hope that this updated catalogue will help our continuing effort of understanding the evolution of blood sucking in vector arthropods and the discovery of novel pharmacologically active compounds.

An unexpected finding of this work was the occurrence of four protein families specific to SG that were probably a product of horizontal transfer from prokaryotic organisms to mosquitoes. Previously, the SGS family was shown to localize specifically in the basal surface of SG cells and may function as a Plasmodium receptor [158]. Here we identify three new families of salivary and possibly secreted proteins (62, 56, and 34 kD) characterized by having uniexonic genes and PSI-BLAST retrieval of only salivary proteins of hematophagous Diptera and bacterial proteins. Although horizontal gene transfer is common in prokaryotic organisms, it is a relatively rare finding in eukaryotes [161]. To the extent that these genes are really of bacterial origin, it may emphasize the unusual paths of SG gene evolution in the quest of hematophagous animals to obtain their 'perfect' potion that allows disarming of the complex host pathways of inflammation and hemostasis that would otherwise disrupt blood feeding.

## Methods

### Mosquitoes

Two laboratory colonies were used in this work, one at Dr. Ribeiro's laboratory at the National Institutes of Health (NIH), and the other in Dr. Wikel's laboratory at the University of Connecticut Health Center (UCHC). Both mosquito colonies were the Liverpool/blackeye strain of *Æ. ægypti*. Insectary rooms were kept at 26°C ± 0.5°C (NIH) or 27°C ± 0.5°C (UCHC), with a relative humidity of 70% to 75% and a 16 h:8 h light:dark photoperiod. Adult female mosquitoes used in the experiments were 0–7 days old, took no blood meals, and were maintained on a diet of 10% Karo syrup solution (NIH) or raisins (UCHC).

### SG isolation

At the NIH, SG from adult female mosquitoes were dissected and transferred to 20μl Hepes saline (HS; NaCl 0.15 M, 10 mM Hepes, pH7.0) in 1.5 ml polypropylene vials in groups of 20 pairs of glands in 20 μl of HS or as individual glands in 10μl of HS. SG were kept at -75°C until needed. At UCHC, adult female mosquitoes were primed for blood feeding before dissecting out their SG by placing a human hand close to the mosquito cage for 3–5 min (without letting the mosquitoes probe). SG were dissected and placed into a solution of 75% RNA-Later (Ambion) 25% 1 × PBS (RNAse free) and stored in 100% RNA-Later at -20°C for isolating polyA+ RNA.

*Æ. ægypti *SG mRNA was isolated from 80SG pairs (NIH) or 110 pairs (UCHC) from adult females at days 1 and 2(NIH) or 1–4 (UCHC) after emergence using the Micro-FastTrack mRNA isolation kit (Invitrogen) (NIH) or the Oligotex™ direct mRNA isolation kit (Qiagen) (UCHC). The PCR-based cDNA library was made following the instructions for the SMART (switching mechanism at 5' end of RNA transcript) cDNA library construction kit (Clontech). This kit provided a method for producing high-quality, full-length cDNA libraries from nanogram quantities of polyA+ or total RNA. It utilizes a specially designed oligonucleotide named SMARTIV™ in the first-strand synthesis to generate high yields of full-length, double-stranded cDNA. *Æ. ægypti *SG polyA+ RNA (300 ng) was used for reverse transcription to cDNA using PowerScript reverse transcriptase (Clontech), the SMARTIV oligonucleotide, and the CDS III/3' primer (Clontech). The reaction was carried out at 42°C for 1 h. Second-strand synthesis was performed by a long-distance PCR-based protocol using the 5' PCR primer and the CDS III/3' primer as sense and antisense primers, respectively. These two primers also create *Sfi1A *and *B*restriction enzyme sites at the end of nascent cDNA. Advantage™ Taq polymerase mix (Clontech) was used to carry out the long-distance PCR reaction on a Perkin Elmer GeneAmp^® ^PCR system9700 (Perkin Elmer Corp.). The PCR conditions were: 95°C for 20s; 24 cycles of 95°C for 5s, 68°C for 6 min. A small portion of the cDNA was analyzed on a 1.1% agarose/EtBr (0.1 μg/ml) gel to check for the quality and range of the cDNA synthesized. Double-stranded cDNA was immediately treated with proteinaseK (0.8 μg/ml) at 45°C for 20 min, extracted with phenol:chloroform:iso-amyl alcohol mixture, and precipitated using sodium acetate (200 mM), glycogen (0.12 μg/ml), and 95% ethanol. The clean double-stranded cDNA was then digested with *SfiI *restriction enzyme at 50°C for 2 h followed by size fractionation on a ChromaSpin-400 drip column (Clontech). The profiles of the fractions were checked on a 1.1% agarose/EtBr (0.1 μg/ml), and fractions containing cDNA of more than 400 bp were pooled and concentrated by precipitation. The cDNA were then ligated into a λ TriplEx2 vector (Clontech), and the resulting ligation mixture was packaged using GigaPack^® ^IIIPlus packaging extract (Stratagene) according to the manufacturer's instructions. The packaged library was plated by infecting log-phase XL1-Blue *Escherichia coli *cells (Clontech). The percentage of recombinant clones was determined by performing a blue-white selection screening on LB/MgSO_4 _plates containing X-gal/IPTG. Recombinants were also determined by PCR, using vector primers (5' λ TriplEx2 and 3' λ TriplEx2 sequencing primers) flanking the inserted cDNA and visualizing the products on a 1.1% agarose/EtBr gel.

### Sequencing of the *Æ. ægypti *cDNA Library

The *Æ. ægypti *SG cDNA library was plated on LB/MgSO_4 _plates containing X-gal/IPTG, to an average of 250 plaques per 150 mm Petri plate. Recombinant (white) plaques were randomly picked up and transferred to 96-well MICROTEST™ U-bottom plates (BD BioSciences) containing 100 μls of SM buffer (0.1 M NaCl, 0.01 M MgSO_4_, 0.035 M Tris-HCl [pH7.5], 0.01% gelatin) per well. The plates were covered and placed on a gyrating shaker for 30 min at room temperature. The phage suspension was either immediately used for PCR or stored at 4°C for future use.

To amplify the cDNA using a PCR reaction, 4 μl of the phage sample was used as a template. The primers were sequences from the λ TriplEx2 vector and named pTEx2 5 seq (5' -TCCGAGATCTGGACGAGC-3' ) and pTEx2 3LD (5' -atacgactcactatagggcgaa ttggc-3' ), positioned at the 5' end and the 3' end of the cDNA insert, respectively. The reaction was carried out in 96-well flexible PCR plates (Fisher Scientific) using TaKaRa EX Taq polymerase (TAKARA; Mirus Bio), on a Perkin Elmer GeneAmp^® ^PCR system9700 (Perkin Elmer Corp.). The PCR conditions were: 1 hold of 95°C for 3 min, 25 cycles of 95°C for 1 min, 61°C for 30s, 72°C for 2 min. The amplified products were analyzed on a 1.5% agarose/EtBr gel. cDNA library clones (1100 clones) were PCR amplified, and those showing a single band were selected for sequencing. Approximately 200–250 ng of each PCR product was transferred to Thermo-Fast 96-well PCR paltes (ABgene Corp.) and frozen at -20°C. Sequencing of the Wikel's laboratory library was performed by Agencourt Bioscience Corp., and a total of 1,017 cDNA library clones was sequenced. The library constructed in Ribeiro's lab was sequenced locally using an 8 capillary CEQ 2000 DNA sequencing instrument (Beckman Coulter, Inc) to provide 2,759 sequences.

### 2D-Gel electrophoresis

2D gel electrophoresis was performed using ZOOM IPGRunner System (Invitrogen) under manufacturer's recommended running conditions. Briefly, approximately 50 μg of sample proteins (approximately 15 pairs of SG) were solubilized with 155 μl rehydration buffer (7 M urea, 2 M thiourea, 2% CHAPS, 20 mM DTT, 0.5% carrier ampholytes, pH3-10). The samples were absorbed by rehydration ZOOM strips (7 cm; pH3-10NL) overnight at room temperature and then focused under manufacturer's recommended conditions. The focused IPG strips were reduced/alkylated/equilibrated with reducing and then alkylation reagents dissolved in the sample buffer. The strips were then applied onto NuPAGE 4–12% Bis-Tris ZOOM gels (Invitrogen). The gels were run under MOPS buffer and stained with SeeBlue staining solution (Bio-Rad). A total of 75 spots were selected for tryptic digestion, based on their staining intensity. The gel picture and 23 protein bands matched to *Æ. ægypti *proteins are shown in Figure 4.

### Protein identification by mass spectrometry

Protein identification of 2Dgel-separated proteins was performed on reduced and alkylated trypsin-digested samples prepared by standard mass spectrometry protocols. Tryptic digests were analyzed by coupling the Nanomate (Advion BioSciences) – an automated chip-based nano-electrospray interface source – to a quadrupole time-of-flight mass spectrometer, QStarXL MS/MS System (Applied Biosystems/Sciex). Computer-controlled, data-dependent automated switching to MS/MS provided peptide sequence information. AnalystQS software (Applied Biosystems/Sciex) was used for data acquisition. Data processing and databank searching were performed with Mascot software (Matrix Science). The NR protein database from the NCBI, National Library of Medicine, NIH, was used for the search analysis, as was a protein database generated during the course of this work.

### Bioinformatic tools and procedures

EST were trimmed of primer and vector sequences, clusterized, and compared with other databases as described before [102]. The BLAST tool [162], CAP3 assembler [163], ClustalW [164], and Treeview software [165] were used to compare, assemble, and align sequences and to visualize alignments. Phylogenetic analysis and statistical neighbor-joining bootstrap tests of the phylogenies were also done with the Mega3 package [166]. For functional annotation of the transcripts we used the tool blastx [107] to compare the nt sequences with the NCBI NR protein database of the NCBI and to the Gene Ontology (GO) database [167]. The tool rpsblast [107] was used to search for conserved protein domains in the Pfam [168], Smart [169], Kog [170], and conserved domains (CDD) databases [171]. We have also compared the transcripts with other subsets of mitochondrial and rRNA nt sequences downloaded from NCBI and to several organism proteomes downloaded from NCBI (yeast), Flybase (*D. melanogaster*), or ENSEMBL (*An. gambiæ*). Segments of the three-frame translations of the EST (as the libraries were unidirectional, we did not use six-frame translations) starting with a methionine in the first 100 predicted aa – or the predicted protein translation, in the case of complete coding sequences – were submitted to the SignalP server [172] to help identify translation products that could be secreted. O-glycosylation sites on the proteins were predicted with the program NetOGlyc [173]. Functional annotation of the transcripts was based on all the comparisons above. Following inspection of all results, transcripts were classified as either (S)ecretory, (H)ousekeeping, or of (U)nknown function, with further subdivisions based on function and/or protein families. To map the EST and contigs in the genome, blastn was used [107]. To speed the program, each genomic fasta file was broken into 30-kb fragments with 5 kb from previous sequence. For visualization of EST on the *Æ. ægypti *genome, we used the Artemis tool [174] after transforming the blastn output to a file compatible to Artemis using a program written in Visual Basic.

To compare the EST frequency in *Æ. ægypti *salivary cDNA libraries with EST frequency in other libraries whose mRNA derive from other sources (downloaded from the NCBI EST database DBEST), all available EST from *Æ. ægypti *plus the EST set from a EST hemocyte library from DrBruce Christensen's laboratory [175] plus our own salivary EST set were pooled to obtain a total of 232,921 EST; these were assembled as described above to create a searchable annotated database of 28,458 contigs and singletons, which is available for browsing at Anobase [10]. The combined EST database thus derives from 29 different EST libraries, 2 of which are from SG of adult female mosquitoes (4,040 from Ribeiro/Wikel laboratories, and 11,585 from Dr. Sergio Verjovski's laboratory); the remainder are from different organs or whole organisms at different developmental stages, or from adult mosquitoes infected or not with different pathogens. Details of these libraries are available at the EST dataset website [10]. From each of these 28,458 contigs, we determined the EST contribution from each of the 29 libraries to the final assembled contigs, thus obtaining for each contig the total salivary and nonsalivary contribution. A χ^2^test was applied to the data set to determine whether a salivary contribution was above or below the null hypothesis of no differential library contribution when the expected EST frequency was above 5, as indicated for the correct use of the test. When the Pvalue was below 0.05, we considered the deviation of equal EST distribution among salivary and remaining libraries as significant.

### RT-PCR expression analysis

For RT-PCR analysis, SG were dissected from adult females 1 to 5 days after emergence and stored at -80°C. Total RNA was extracted from female glands, carcasses (i.e. adult females with SG removed), and adult males using the TRIZOL reagent (Invitrogen).

Approximately 50 ng RNAse-free DNase-treated total RNA (Invitrogen) was used for the RT-PCR amplification by the SuperScript one-step RT-PCR system (Invitrogen) according to manufacturer's instructions. Typically, reverse transcription (50°C, 30 min) and heat inactivation of the reverse transcriptase (94°C, 2 min) were followed by 30 PCR cycles: 30s at 94°C, 30s at 55°C, 1 min. at 72°C; 25 cycles were used for the amplification of the ribosomal protein S5 mRNA (*rpS5*) to keep the reaction below saturation levels and to allow reliable normalization. For some clones (gi|94468620, gi|94468350, gi|94468634, and gi|42632615), 35 cycles of amplification were needed to obtain detectable bands. The oligonucleotide primers used for *rpS5 *amplification were: rpS5-F, 5' -ATTACATCGCCGTCAAGG AG-3' , and rpS5-R, 5' -TCATC ATCAGCGAGTTGGTC-3'. The sequence of the other oligonucleotide primers is available as Supplemental Material. Amplification reactions were analyzed on 1.2% agarose gels. Each sample was analyzed by RT-PCR two to three times using independent batches of total RNA.

## Abbreviations

2D, two dimensional; aa, amino acid; ADA, adenosine deaminase; AMP, antimicrobial peptide; AG5, antigen-5 family; EST, expressed sequence tag; Hclass, housekeeping; HS, Hepes saline; kbase, kilobase; HS, Hepes saline; kb, kilobase; NR, nonredundant; nt, nucleotide; OBP, odorant-binding protein; PNase, purine nucleosidase; PPO, prophenyloxidase; RT-PCR, reverse transcriptase polymerase chain reaction; Sclass, secreted; SDS-PAGE, sodium dodecyl sulfate/polyacrylamide gel electrophoresis; SG, salivary gland; SMART, switching mechanism at 5' end of RNA transcript; Tclass, transposable element; Uclass, unknown function.

## Authors' contributions

JMCR performed data analysis, supervised sequencing the NIH library, and contributed to the manuscript. BAsupervised tissue expression experiments, performed data analysis and contributed to the manuscript. FLperformed tissue expression experiments and data analysis, and contributed to the manuscript. EChelped with library sequencing at NIH and proteome analysis, and contributed to the manuscript. VMPparticipated in sequencing the NIH library. PKCparticipated in sequencing the UCHC library, analyzed data, and contributed to the manuscript. SKWsupervised sequencing the UCHC library, analyzed data, and contributed to the manuscript.

## Supplementary Material

Additional file 1 Supplemental Table S1. Click here for file

Additional file 2 Supplemental Table S2. Click here for file

## References

[B1] Ribeiro JMC (1987). Role of arthropod saliva in blood feeding. Ann Rev Entomol.

[B2] Ribeiro JMC (1995). Blood-feeding arthropods: Live syringes or invertebrate pharmacologists?. Infect Agents Dis.

[B3] Valenzuela JG, Pham VM, Garfield MK, Francischetti IM, Ribeiro JMC (2002). Toward a description of the sialome of the adult female mosquito Aedes aegypti. Insect Biochem Mol Biol.

[B4] Transposable element similar to Strongylocentrotus TE. http://www.ncbi.nlm.nih.gov/sutils/blink.cgi?pid=72011940.

[B5] Tc1-like transposase. http://www.ncbi.nlm.nih.gov/sutils/blink.cgi?pid=76154387.

[B6] Updated Aedes aegypti sialome - Supplemental Table 1. http://www.ncbi.nlm.nih.gov/projects/omes/Ae_aegypti_sialome_2006/Sup_table_1/AE-Sup-table1.xls.

[B7] Zimmermann B, Dames P, Walz B, Baumann O (2003). Distribution and serotonin-induced activation of vacuolar-type H+-ATPase in the salivary glands of the blowfly Calliphora vicina. J Exp Biol.

[B8] Ribeiro JMC (2006). Updated Aedes aegypti Sialome - Supplemental Table 1.

[B9] Ribeiro JM, Topalis P, Louis C (2004). AnoXcel: an Anopheles gambiae protein database. Insect Mol Biol.

[B10] Anobase site url. http://www.anobase.org.

[B11] Aedes updated sialome - Supplemental Table 2. http://www.ncbi.nlm.nih.gov/projects/omes/Ae_aegypti_sialome_2006/Sup_table_2/Ae-Sup-table2.xls.

[B12] Arca B, Lombardo F, Valenzuela JG, Francischetti IM, Marinotti O, Coluzzi M, Ribeiro JM (2005). An updated catalogue of salivary gland transcripts in the adult female mosquito, Anopheles gambiae. J Exp Biol.

[B13] Marinotti O, Calvo E, Nguyen QK, Dissanayake S, Ribeiro JM, James AA (2006). Genome-wide analysis of gene expression in adult Anopheles gambiae. Insect Mol Biol.

[B14] James AA, Blackmer K, Marinotti O, Ghosn CR, Racioppi JV (1991). Isolation and characterization of the gene expressing the major salivary gland protein of the female mosquito, Aedes aegypti. Mol Biochem Parasitol.

[B15] Valenzuela JG, Charlab R, Gonzalez EC, Miranda-Santos IKF, Marinotti O, Francischetti IM, Ribeiro JMC (2002). The D7 family of salivary proteins in blood sucking Diptera. Insect Mol Biol.

[B16] Arca B, Lombardo F, Lanfrancotti A, Spanos L, Veneri M, Louis C, Coluzzi M (2002). A cluster of four D7-related genes is expressed in the salivary glands of the African malaria vector Anopheles gambiae. Insect Mol Biol.

[B17] Hekmat-Scafe DS, Dorit RL, Carlson JR (2000). Molecular evolution of odorant-binding protein genes OS-E and OS-F in Drosophila. Genetics.

[B18] Calvo E, Mans BJ, Andersen JF, Ribeiro JM (2005). Function and evolution of a mosquito salivary protein family. J Biol Chem.

[B19] Isawa H, Yuda M, Orito Y, Chinzei Y (2002). A mosquito salivary protein inhibits activation of the plasma contact system by binding to factor XII and high molecular weight kininogen. J Biol Chem.

[B20] Campbell CL, Vandyke KA, Letchworth GJ, Drolet BS, Hanekamp T, Wilson WC (2005). Midgut and salivary gland transcriptomes of the arbovirus vector Culicoides sonorensis (Diptera: Ceratopogonidae). Insect Mol Biol.

[B21] Updated sialome of Anopheles gambiae - Figure 4. http://www.ncbi.nlm.nih.gov/projects/omes/An_gambiae_sialome-2005/Fig4.pdf.

[B22] Rai KS, Black WC (1999). Mosquito genomes: Structure, organization, and evolution. Adv Genet.

[B23] Warren AM, Crampton JM (1991). The Aedes aegypti genome: complexity and organization. Genet Res.

[B24] Koonin EV (2005). Orthologs, Paralogs, and Evolutionary Genomics. Annu Rev Genet.

[B25] Nei M, Rooney AP (2005). Concerted and birth-and-death evolution of multigene families. Annu Rev Genet.

[B26] Hengst U, Albrecht H, Hess D, Monard D (2001). The phosphatidylethanolamine-binding protein is the prototype of a novel family of serine protease inhibitors. J Biol Chem.

[B27] Simister PC, Banfield MJ, Brady RL (2002). The crystal structure of PEBP-2, a homologue of the PEBP/RKIP family. Acta Crystallogr D Biol Crystallogr.

[B28] Stark KR, James AA (1995). A factor Xa-directed anticoagulant from the salivary glands of the yellow fever mosquito Aedes aegypti. Exp Parasitol.

[B29] Stark KR, James AA (1998). Isolation and characterization of the gene encoding a novel factor Xa-directed anticoagulant from the yellow fever mosquito, Aedes aegypti. J Biol Chem.

[B30] Aedes aegypti updated sialome - Serpin1. http://www.ncbi.nlm.nih.gov/projects/omes/Ae_aegypti_sialome_2006/Sup_table_2/links/NR/AE-114-NR.txt.

[B31] Aedes aegypti updated sialome - link to serpins in A. albopictus. http://www.ncbi.nlm.nih.gov/projects/omes/Ae_aegypti_sialome_2006/Sup_table_2/links/NR/18568304-NR.txt.

[B32] Ribeiro JMC (1992). Characterization of vasodilator from the salivary glands of the Yellow Fever mosquito, Aedes aegypti. J Exp Biol.

[B33] Champagne D, Ribeiro JMC (1994). Sialokinins I and II: Two salivary tachykinins from the Yellow Fever mosquito, Aedes aegypti. Proc Natl Acad Sci (USA).

[B34] Beerntsen BT, Champagne DE, Coleman JL, Campos YA, James AA (1999). Characterization of the Sialokinin I gene encoding the salivary vasodilator of the yellow fever mosquito, Aedes aegypti. Insect Mol Biol.

[B35] Champagne DE, Smartt CT, Ribeiro JM, James AA (1995). The salivary gland-specific apyrase of the mosquito Aedes aegypti is a member of the 5'-nucleotidase family. Proc Natl Acad Sci U S A.

[B36] Ribeiro JM, Charlab R, Valenzuela JG (2001). The salivary adenosine deaminase activity of the mosquitoes Culex quinquefasciatus and Aedes aegypti. J Exp Biol.

[B37] Ribeiro JM, Valenzuela JG (2003). The salivary purine nucleosidase of the mosquito, Aedes aegypti. Insect Biochem Mol Biol.

[B38] Charlab R, Valenzuela JG, Rowton ED, Ribeiro JM (1999). Toward an understanding of the biochemical and pharmacological complexity of the saliva of a hematophagous sand fly Lutzomyia longipalpis. Proc Natl Acad Sci U S A.

[B39] 5'Nucleotidase comparison to Aedes. http://www.ncbi.nlm.nih.gov/blast/bl2seq/wblast2.cgi?one=94469274&two=763502&prot=blastp&expect=300.

[B40] 5' nucleotidase comparison to Culex. http://www.ncbi.nlm.nih.gov/blast/bl2seq/wblast2.cgi?one=94469274&two=38350627&prot=blastp&expect=300.

[B41] Misumi Y, Ogata S, Ohkubo K, Hirose S, Ikehara Y (1990). Primary structure of human placental 5'-nucleotidase and identification of the glycolipid anchor in the mature form. Eur JBiochem.

[B42] Ogata S, Hayashi Y, Misumi Y, Ikehara Y (1990). Membrane-anchoring domain of rat liver 5'-nucleotidase: identification of the COOH-terminal serine-523 covalently attached with a glycolipid. Biochemistry.

[B43] Serrano AA, Schenkman SYN, Mehlert A, Richardson JM, Ferguson MAJ (1995). The lipid structure of the glycosylphosphatidylinositol-anchored mucin-like sialic acid acceptors of Trypanosoma cruzi changes during parasite differentiation from epimastigotes to infective metacyclic trypomastigote forms. J Biol Chem.

[B44] Ribonuclease of the T2 family. http://www.ncbi.nlm.nih.gov/sutils/blink.cgi?pid=94468968.

[B45] Deshpande RA, Shankar V (2002). Ribonucleases from T2 family. Crit Rev Microbiol.

[B46] AEA-876 sequence. http://www.ncbi.nlm.nih.gov/entrez/viewer.fcgi?db=protein&val=94468664.

[B47] AEA-562 sequence. http://www.ncbi.nlm.nih.gov/entrez/viewer.fcgi?db=protein&val=94468658.

[B48] CUB Domain in PFAM. http://www.sanger.ac.uk/cgi-bin/Pfam/getacc?PF00431.

[B49] Clip domain serine protease. http://www.ncbi.nlm.nih.gov/sutils/blink.cgi?pid=18568334.

[B50] AE-226 sequence. http://www.ncbi.nlm.nih.gov/sutils/blink.cgi?pid=94468410.

[B51] Aedes aegypti amylase. http://www.ncbi.nlm.nih.gov/sutils/blink.cgi?pid=2190949.

[B52] Grossman GL, James AA (1993). The salivary glands of the vector mosquito, Aedes aegypti, express a novel member of the amylase gene family. Insect Mol Biol.

[B53] Aedes aegypti maltase. http://www.ncbi.nlm.nih.gov/sutils/blink.cgi?pid=84861.

[B54] James AA, Blackmer K, Racioppi JV (1989). A salivary gland-specific, maltase-like gene of the vector mosquito, Aedes aegypti. Gene.

[B55] Aedes aegypti salivary phosphatase. http://www.ncbi.nlm.nih.gov/sutils/blink.cgi?pid=94469242.

[B56] Aedes aegypti esterase. http://www.ncbi.nlm.nih.gov/sutils/blink.cgi?pid=18568286.

[B57] Argentine JA, James AA (1995). Characterization of a salivary gland-specific esterase in the vector mosquito, Aedes aegypti. Insect Biochem Mol Biol.

[B58] Aedes aegypti salivary lysozyme. http://www.ncbi.nlm.nih.gov/sutils/blink.cgi?pid=18568288.

[B59] Rossignol PA, Lueders AM (1986). Bacteriolytic factor in the salivary glands of  Aedes aegypti. Comp Biochem Physiol.

[B60] Marinotti O, James A, Ribeiro JMC (1990). Diet and salivation in female Aedes aegypti mosquitoes. J Insect Physiol.

[B61] Moreira-Ferro CK, Marinotti O, Bijovsky AT (1999). Morphological and biochemical analyses of the salivary glands of the malaria vector, Anopheles darlingi. Tissue Cell.

[B62] Vasta GR, Ahmed H, Fink NE, Elola MT, Marsh AG, Snowden A, Odom EW (1994). Animal lectins as self/non-self recognition molecules. Biochemical and genetic approaches to understanding their biological roles and evolution. Ann N Y Acad Sci.

[B63] Osta MA, Christophides GK, Kafatos FC (2004). Effects of mosquito genes on Plasmodium development. Science.

[B64] Arocas V, Castro HC, Zingali RB, Guillin MC, Jandrot-Perrus M, Bon C, Wisner A (1997). Molecular cloning and expression of bothrojaracin, a potent thrombin inhibitor from snake venom. Eur J Biochem.

[B65] Polgar J, Clemetson JM, Kehrel BE, Wiedemann M, Magnenat EM, Wells TNC, Clemetson KJ (1997). Platelet activation and signal transduction by convulxin, a C-type lectin from Crotalus durissus terrificus (tropical rattlesnake) venom via the p62/GPVI collagen receptor. J Biol Chem.

[B66] Vasta GR, Quesenberry M, Ahmed H, O'Leary N (1999). C-type lectins and galectins mediate innate and adaptive immune functions: their roles in the complement activation pathway. Dev Comp Immunol.

[B67] Metcalf RL (1945). The physiology of the salivary glands of Anopheles quadrimaculatus. J Nat Malaria Soc.

[B68] Vaughan JA, Noden BH, Beier JC (1991). Concentration of human erythrocytes by anopheline mosquitoes (Diptera:Culicidae) during feeding. J Med Entomol.

[B69] Imaginal disk growth factor. http://www.ncbi.nlm.nih.gov/entrez/viewer.fcgi?val=56684623.

[B70] Bacteria responsive protein. http://www.ncbi.nlm.nih.gov/entrez/viewer.fcgi?val=46095203.

[B71] Bacteria responsive protein 2. http://www.ncbi.nlm.nih.gov/entrez/viewer.fcgi?val=46095205.

[B72] Shi L, Paskewitz SM (2004). Identification and molecular characterization of two immune-responsive chitinase-like proteins from Anopheles gambiae. Insect Mol Biol.

[B73] Fibrinogen C motif on PFAM.

[B74] Matsushita M, Fujita T (2002). The role of ficolins in innate immunity. Immunobiology.

[B75] Wang X, Zhao Q, Christensen BM (2005). Identification and characterization of the fibrinogen-like domain of fibrinogen-related proteins in the mosquito, Anopheles gambiae, and the fruitfly, Drosophila melanogaster, genomes. BMC Genomics.

[B76] Salivary gambicin. http://www.ncbi.nlm.nih.gov/sutils/blink.cgi?pid=18568310.

[B77] Aedes aegypti defensin. http://www.ncbi.nlm.nih.gov/sutils/blink.cgi?pid=48256697.

[B78] AET-590 Blast results. http://www.ncbi.nlm.nih.gov/blast/bl2seq/wblast2.cgi?one=94469250&two=57918801&prot=blastp&expect=300.

[B79] Couillault C, Pujol N, Reboul J, Sabatier L, Guichou JF, Kohara Y, Ewbank JJ (2004). TLR-independent control of innate immunity in Caenorhabditis elegans by the TIR domain adaptor protein TIR-1, an ortholog of human SARM. Nat Immunol.

[B80] AET-462 sequence. http://www.ncbi.nlm.nih.gov/entrez/viewer.fcgi?db=protein&val=94469086.

[B81] AET-11358 sequence. http://www.ncbi.nlm.nih.gov/entrez/viewer.fcgi?db=protein&val=94468690.

[B82] Rothstein DM, Helmerhorst EJ, Spacciapoli P, Oppenheim FG, Friden P (2002). Histatin-derived peptides: potential agents to treat localised infections. Expert Opin Emerg Drugs.

[B83] Lai R, Takeuchi H, Lomas LO, Jonczy J, Rigden DJ, Rees HH, Turner PC (2004). A new type of antimicrobial protein with multiple histidines from the hard tick, Amblyomma hebraeum. Faseb J.

[B84] AEA-233 sequence. http://www.ncbi.nlm.nih.gov/sutils/blink.cgi?pid=94468652.

[B85] i23R sequence. http://www.ncbi.nlm.nih.gov/entrez/viewer.fcgi?val=AAK55497.1.

[B86] Morlais I, Mori A, Schneider JR, Severson DW (2003). A targeted approach to the identification of candidate genes determining susceptibility to Plasmodium gallinaceum in Aedes aegypti. Mol Genet Genomics.

[B87] Gram negative binding protein. http://www.ncbi.nlm.nih.gov/sutils/blink.cgi?pid=18568294.

[B88] AE-7210 blast comparison. http://www.ncbi.nlm.nih.gov/sutils/blink.cgi?pid=94468610.

[B89] Megraw T, Kaufman TC, Kovalick GE (1998). Sequence and expression of Drosophila Antigen 5-related 2, a new member of the CAP gene family. Gene.

[B90] Francischetti IM, Valenzuela JG, Pham VM, Garfield MK, Ribeiro JM (2002). Toward a catalog for the transcripts and proteins (sialome) from the salivary gland of the malaria vector Anopheles gambiae. J Exp Biol.

[B91] Li S, Kwon J, Aksoy S (2001). Characterization of genes expressed in the salivary glands of the tsetse fly, Glossina morsitans morsitans. Insect Mol Biol.

[B92] Milne TJ, Abbenante G, Tyndall JD, Halliday J, Lewis RJ (2003). Isolation and characterization of a cone snail protease with homology to CRISP proteins of the pathogenesis-related protein superfamily. J Biol Chem.

[B93] Yamazaki Y, Koike H, Sugiyama Y, Motoyoshi K, Wada T, Hishinuma S, Mita M, Morita T (2002). Cloning and characterization of novel snake venom proteins that block smooth muscle contraction. Eur JBiochem.

[B94] Yamazaki Y, Morita T (2004). Structure and function of snake venom cysteine-rich secretory proteins. Toxicon.

[B95] Nobile M, Noceti F, Prestipino G, Possani LD (1996). Helothermine, a lizard venom toxin, inhibits calcium current in cerebellar granules. Exp Brain Res.

[B96] AE-796 sequence. http://www.ncbi.nlm.nih.gov/entrez/viewer.fcgi?val=94468620.

[B97] PAN_AP_HGF domain. http://www.ncbi.nlm.nih.gov/projects/omes/Ae_aegypti_sialome_2006/Sup_table_2/links/CDD/AE-796-CDD.txt.

[B98] AE-389 blast comparison. http://www.ncbi.nlm.nih.gov/sutils/blink.cgi?pid=94468538.

[B99] TIL domain on AE-389. http://www.ncbi.nlm.nih.gov/projects/omes/Ae_aegypti_sialome_2006/Sup_table_2/links/CDD/AE-389-CDD.txt.

[B100] TIL domain peptide in An. stephensi. http://www.ncbi.nlm.nih.gov/Structure/cdd/wrpsb.cgi?INPUT_TYPE=precalc&SEQUENCE=27372905.

[B101] TIL domain peptide in An. gambiae. http://www.ncbi.nlm.nih.gov/Structure/cdd/wrpsb.cgi?INPUT_TYPE=precalc&SEQUENCE=87080403.

[B102] Valenzuela JG, Francischetti IM, Pham VM, Garfield MK, Ribeiro JM (2003). Exploring the salivary gland transcriptome and proteome of the Anopheles stephensi mosquito. Insect Biochem Mol Biol.

[B103] Calvo E, Pham VM, Lombardo F, Arca B, Ribeiro JM (2006). The sialotranscriptome of adult male Anopheles gambiae mosquitoes. Insect Biochem Mol Biol.

[B104] Ixodidin sequence. http://www.ncbi.nlm.nih.gov/Structure/cdd/wrpsb.cgi?INPUT_TYPE=precalc&SEQUENCE=73620968.

[B105] Fogaca AC, Almeida IC, Eberlin MN, Tanaka AS, Bulet P, Daffre S (2005). Ixodidin, a novel antimicrobial peptide from the hemocytes of the cattle tick Boophilus microplus with inhibitory activity against serine proteinases. Peptides.

[B106] Blast comparisons of 56 kDa proteins. http://www.ncbi.nlm.nih.gov/projects/omes/Ae_aegypti_sialome_2006/Sup_table_2/links/NR/18568292-NR.txt.

[B107] Altschul SF, Madden TL, Schaffer AA, Zhang J, Zhang Z, Miller W, Lipman DJ (1997). Gapped BLAST and PSI-BLAST: a new generation of protein database search programs. Nucleic Acids Res.

[B108] PSI-Blast of 56 kDa proteins. http://www.ncbi.nlm.nih.gov/projects/omes/Ae_aegypti_sialome_2006/Sup_table_2/links/PSIB/56kda-psi.rtf.

[B109] Single exon structure of 56 kDa protein. http://www.ncbi.nlm.nih.gov/projects/omes/Ae_aegypti_sialome_2006/Sup_table_2/links/AEGFRAG/18568292-AEGFRAG.txt.

[B110] Single exon of An. gambiae 56 kDa protein. http://www.ncbi.nlm.nih.gov/projects/omes/An_gambiae_sialome-2005/table4/links/hyp55.3-AGFRAG.txt.

[B111] Three exon structure of 41 kDa family. http://www.ncbi.nlm.nih.gov/projects/omes/Ae_aegypti_sialome_2006/Sup_table_2/links/AEGFRAG/AET-2909-AEGFRAG.txt.

[B112] 41 kDa family mismatch. http://www.ncbi.nlm.nih.gov/sutils/blink.cgi?pid=61742035.

[B113] PSIBlast of 41 kDa family. http://www.ncbi.nlm.nih.gov/projects/omes/Ae_aegypti_sialome_2006/Sup_table_2/links/PSIB/41kda-psi.rtf.

[B114] Simons FE, Peng Z (2001). Mosquito allergy: recombinant mosquito salivary antigens for new diagnostic tests. Int Arch Allergy Immunol.

[B115] 30 kDA allele 1. http://www.ncbi.nlm.nih.gov/entrez/viewer.fcgi?val=14423642.

[B116] 30 kDa allele 2. http://www.ncbi.nlm.nih.gov/entrez/viewer.fcgi?val=18568322.

[B117] 29 kDa family transcripts. http://www.ncbi.nlm.nih.gov/projects/omes/Ae_aegypti_sialome_2006/Sup_table_2/links/ae-comp-ext95-60-Sim-CLTL7.txt.

[B118] Non-salivary 29 kDa family member. http://www.ncbi.nlm.nih.gov/projects/omes/Ae_aegypti_sialome_2006/Sup_table_2/links/AE-EST/AE-236A-AE-EST.txt.

[B119] PSIBlast of 29 kDa family. http://www.ncbi.nlm.nih.gov/projects/omes/Ae_aegypti_sialome_2006/Sup_table_2/links/PSIB/29kda-psi.rtf.

[B120] AE-212 blast comparison. http://www.ncbi.nlm.nih.gov/projects/omes/Ae_aegypti_sialome_2006/Sup_table_2/links/NR/AE-212-NR.txt.

[B121] AE-165 sequence. http://www.ncbi.nlm.nih.gov/sutils/blink.cgi?pid=94468362.

[B122] AE-163 sequence. http://www.ncbi.nlm.nih.gov/sutils/blink.cgi?pid=94468360.

[B123] Blast comparison of AE-165. http://www.ncbi.nlm.nih.gov/projects/omes/Ae_aegypti_sialome_2006/Sup_table_2/links/NR/AE-165-NR.txt.

[B124] Aedes protein similar to An. gambiae sSG8. http://www.ncbi.nlm.nih.gov/projects/omes/Ae_aegypti_sialome_2006/Sup_table_2/links/NR/AE-196-NR.txt.

[B125] Aedes protein similar to W rich Culex protein. http://www.ncbi.nlm.nih.gov/projects/omes/Ae_aegypti_sialome_2006/Sup_table_2/links/NR/61742023-NR.txt.

[B126] Aedes protein similar to Culex salivary protein. http://www.ncbi.nlm.nih.gov/projects/omes/Ae_aegypti_sialome_2006/Sup_table_2/links/NR/AE-209-NR.txt.

[B127] Aedes protein similar to Anopheles protein. http://www.ncbi.nlm.nih.gov/projects/omes/Ae_aegypti_sialome_2006/Sup_table_2/links/AGPROT/AE-225-AGPROT.txt.

[B128] Similar to Drosophila peptide. http://www.ncbi.nlm.nih.gov/projects/omes/Ae_aegypti_sialome_2006/Sup_table_2/links/DMPROT/AE-225-DMPROT.txt.

[B129] Single exon structure of 62 kDa protein. http://www.ncbi.nlm.nih.gov/projects/omes/Ae_aegypti_sialome_2006/Sup_table_2/links/AEGFRAG/18568302-AEGFRAG.txt.

[B130] 62 kDa homologue in Ae. albopictus. http://www.ncbi.nlm.nih.gov/sutils/blink.cgi?pid=18568300.

[B131] 62 kDa family similarity to myosin. http://www.ncbi.nlm.nih.gov/projects/omes/Ae_aegypti_sialome_2006/Sup_table_2/links/KOG/18568302-KOG.txt.

[B132] Two exon structure of a 34 kDa family member. http://www.ncbi.nlm.nih.gov/projects/omes/Ae_aegypti_sialome_2006/Sup_table_2/links/AEGFRAG/AEA-286-AEGFRAG.txt.

[B133] Single exon structure of 34 kDa family members. http://www.ncbi.nlm.nih.gov/projects/omes/Ae_aegypti_sialome_2006/Sup_table_2/links/AEGFRAG/AEA-105-AEGFRAG.txt.

[B134] 34 kDa family in Ae. albopictus. http://www.ncbi.nlm.nih.gov/projects/omes/Ae_aegypti_sialome_2006/Sup_table_2/links/NR/18568296-NR.txt.

[B135] 30.5 kDa family member 1 in Aedes albopictus. http://www.ncbi.nlm.nih.gov/projects/omes/Ae_aegypti_sialome_2006/Sup_table_2/links/NR/AE-260-NR.txt.

[B136] Second 30.5 kDa member in Aedes albopictus. http://www.ncbi.nlm.nih.gov/projects/omes/Ae_aegypti_sialome_2006/Sup_table_2/links/NR/61742033-NR.txt.

[B137] 9 kDa family. http://www.ncbi.nlm.nih.gov/projects/omes/Ae_aegypti_sialome_2006/Sup_table_2/links/ae-comp-ext70-60-Sim-CLTL21.txt.

[B138] 9 kDa family in Aedes albopictus. http://www.ncbi.nlm.nih.gov/projects/omes/Ae_aegypti_sialome_2006/Sup_table_2/links/NR/AE-216-NR.txt.

[B139] AE-376 sequence. http://www.ncbi.nlm.nih.gov/sutils/blink.cgi?pid=94468530.

[B140] AE-156 blast comparison. http://www.ncbi.nlm.nih.gov/sutils/blink.cgi?pid=94468356.

[B141] gi|18568314 blast comparison. http://www.ncbi.nlm.nih.gov/sutils/blink.cgi?pid=18568314.

[B142] gi|18568282 sequence. http://www.ncbi.nlm.nih.gov/entrez/viewer.fcgi?db=protein&val=18568282.

[B143] AE-211 sequence. http://www.ncbi.nlm.nih.gov/entrez/viewer.fcgi?db=protein&val=94468390.

[B144] AE-214 sequence. http://www.ncbi.nlm.nih.gov/entrez/viewer.fcgi?db=protein&val=94468394.

[B145] Nelson N, Perzov N, Cohen A, Hagai K, Padler V, Nelson H (2000). The cellular biology of proton-motive force generation by V-ATPases. J Exp Biol.

[B146] Wieczorek H, Huss M, Merzendorfer H, Reineke S, Vitavska O, Zeiske W (2003). The insect plasma membrane H+ V-ATPase: intra-, inter-, and supramolecular aspects. J Bioenerg Biomembr.

[B147] Pullikuth AK, Filippov V, Gill SS (2003). Phylogeny and cloning of ion transporters in mosquitoes. J Exp Biol.

[B148] Novak MG, Ribeiro JMC, Hildebrand JG (1995). 5-Hydroxytriptamine in the salivaryt glands of adult female Aedes aegypti and its role in regulation of salivation. J Exp Biol.

[B149] Cytochrome P450 in salivary glands. http://www.ncbi.nlm.nih.gov/projects/omes/Ae_aegypti_sialome_2006/Sup_table_2/links/NR/AET-11328-NR.txt.

[B150] Simpson AE (1997). The cytochrome P450 4 (CYP4) family. Gen Pharmacol.

[B151] Peroxisomal protein. http://www.ncbi.nlm.nih.gov/projects/omes/Ae_aegypti_sialome_2006/Sup_table_2/links/KOG/AET-6749-KOG.txt.

[B152] Kynurenine formamidase. http://www.ncbi.nlm.nih.gov/projects/omes/Ae_aegypti_sialome_2006/Sup_table_2/links/NR/AET-12468-NR.txt.

[B153] KOG motif for kynurenine formamidase. http://www.ncbi.nlm.nih.gov/projects/omes/Ae_aegypti_sialome_2006/Sup_table_2/links/KOG/AET-12468-KOG.txt.

[B154] Hirai M, Wang J, Yoshida S, Ishii A, Matsuoka H (2001). Characterization and identification of exflagellation-inducing factor in the salivary gland of Anopheles stephensi (Diptera: Culicidae). Biochem Biophys Res Commun.

[B155] Billker O, Lindo V, Panico M, Etienne AE, Paxton T, Dell A, Rogers M, Sinden RE, Morris HR (1998). Identification of xanthurenic acid as the putative inducer of malaria development in the mosquito. Nature.

[B156] Garcia GE, Wirtz RA, Barr JR, Woolfitt A, Rosenberg R (1998). Xanthurenic acid induces gametogenesis in Plasmodium, the malaria parasite. J Biol Chem.

[B157] Beerntsen BT, Li J (2006). Plasmodium development in white-eye (kh(w)) and transformed strains (kh43) of Aedes aegypti (Diptera: Culicidae). J Med Entomol.

[B158] Korochkina S, Barreau C, Pradel G, Jeffery E, Li J, Natarajan R, Shabanowitz J, Hunt D, Frevert U, Vernick KD (2006). A mosquito-specific protein family includes candidate receptors for malaria sporozoite invasion of salivary glands. Cell Microbiol.

[B159] Intronless structrure of SGS1. http://www.ncbi.nlm.nih.gov/projects/omes/Ae_aegypti_sialome_2006/Sup_table_2/links/AEGFRAG/66828491-AEGFRAG.txt.

[B160] Galperin MY, Koonin EV (2004). 'Conserved hypothetical' proteins: prioritization of targets for experimental study. Nucleic Acids Res.

[B161] Ochman H, Lawrence JG, Groisman EA (2000). Lateral gene transfer and the nature of bacterial innovation. Nature.

[B162] Altschul SF, Gish W (1996). Local alignment statistics. Methods Enzymol.

[B163] Huang X, Madan A (1999). CAP3: A DNA sequence assembly program. Genome Res.

[B164] Thompson JD, Higgins DG, Gibson TJ (1994). CLUSTAL W: improving the sensitivity of progressive multiple sequence alignment through sequence weighting, position-specific gap penalties and weight matrix choice. Nucleic Acids Res.

[B165] Page RD (1996). TreeView: an application to display phylogenetic trees on personal computers. Comput Appl Biosci.

[B166] Kumar S, Tamura K, Nei M (2004). MEGA3: Integrated software for Molecular Evolutionary Genetics Analysis and sequence alignment. Brief Bioinform.

[B167] Ashburner M, Ball CA, Blake JA, Botstein D, Butler H, Cherry JM, Davis AP, Dolinski K, Dwight SS, Eppig JT, Harris MA, Hill DP, Issel-Tarver L, Kasarskis A, Lewis S, Matese JC, Richardson JE, Ringwald M, Rubin GM, Sherlock G (2000). Gene ontology: tool for the unification of biology. The Gene Ontology Consortium. Nat Genet.

[B168] Bateman A, Birney E, Durbin R, Eddy SR, Howe KL, Sonnhammer EL (2000). The Pfam protein families database. Nucleic Acids Res.

[B169] Schultz J, Copley RR, Doerks T, Ponting CP, Bork P (2000). SMART: a web-based tool for the study of genetically mobile domains. Nucleic Acids Res.

[B170] Tatusov RL, Fedorova ND, Jackson JD, Jacobs AR, Kiryutin B, Koonin EV, Krylov DM, Mazumder R, Mekhedov SL, Nikolskaya AN, Rao BS, Smirnov S, Sverdlov AV, Vasudevan S, Wolf YI, Yin JJ, Natale DA (2003). The COG database: an updated version includes eukaryotes. BMC Bioinformatics.

[B171] Marchler-Bauer A, Panchenko AR, Shoemaker BA, Thiessen PA, Geer LY, Bryant SH (2002). CDD: a database of conserved domain alignments with links to domain three-dimensional structure. Nucleic Acids Res.

[B172] Nielsen H, Engelbrecht J, Brunak S, von Heijne G (1997). Identification of prokaryotic and eukaryotic signal peptides and prediction of their cleavage sites. Protein Eng.

[B173] Hansen JE, Lund O, Tolstrup N, Gooley AA, Williams KL, Brunak S (1998). NetOglyc: prediction of mucin type O-glycosylation sites based on sequence context and surface accessibility. Glycoconj J.

[B174] Berriman M, Rutherford K (2003). Viewing and annotating sequence data with Artemis. Brief Bioinform.

[B175] EST hemocyte library from Christensen's laboratory. http://www.ahabs.wisc.edu/~christensenlab/HemocyteEST/asap.html.

